# A ratiometric two-photon probe for quantitative imaging of mitochondrial pH values†
†Electronic supplementary information (ESI) available: Synthesis, additional methods, and figures (Fig. S1–S14). See DOI: 10.1039/c5sc03708e


**DOI:** 10.1039/c5sc03708e

**Published:** 2015-10-27

**Authors:** Avik Ranjan Sarkar, Cheol Ho Heo, Lei Xu, Hyo Won Lee, Ho Young Si, Ji Won Byun, Hwan Myung Kim

**Affiliations:** a Department of Chemistry and Department of Energy Systems Research , Ajou University , Suwon 443-749 , Korea . Email: kimhm@ajou.ac.kr

## Abstract

Mitochondrial pH (pH_mito_) is known to be alkaline (near 8.0) and has emerged as a potential factor for mitochondrial function and disorder. We have developed a ratiometric two-photon probe (**CMP1**) for quantitative analysis of pH_mito_ in live cells and tissues. This probe is designed to function by controlling the intramolecular charge transfer from 2-naphthol, having an ideal p*K*_a_ value (7.86 ± 0.05) in the cells to monitor pH_mito_. This transition results in a marked yellow to red emission color change in response to pH alterations from 6.0 to 9.0. **CMP1** exhibits easy loading, selective and robust staining ability of mitochondria, low cytotoxicity, and bright two-photon excited fluorescence *in situ*, thereby allowing quantitative imaging of the pH_mito_ in live cells and tissues. The ratiometric TPM imaging clearly reveals that subcellular distribution of the pH_mito_ values is heterogeneous, with the pH_mito_ values in the perinuclear region being higher than those at the periphery of the cells. The changes of pH_mito_ values on carbonyl cyanide *m*-chlorophenyl hydrazone (CCCP) treatment and autophagic processes were also investigated along with their morphological alterations at specific subcellular positions. We also used **CMP1** to visualize the pH_mito_ values of Parkinson's disease model astrocytes as well as living hippocampal tissues. Our results demonstrate that **CMP1** will be useful as a quantitative imaging probe to study pH_mito_ in biomedical research.

## Abstract 2

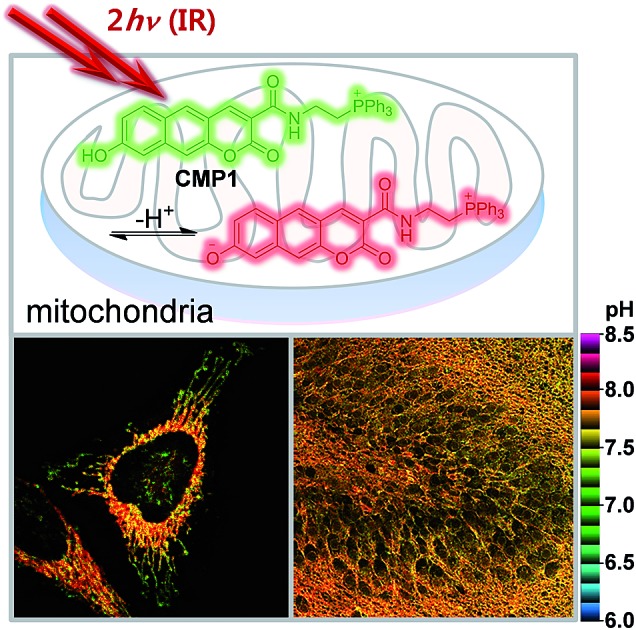
A ratiometric two-photon fluorescent probe for quantitative imaging of mitochondrial pH values in live cells and tissues was reported.

## Introduction

The mitochondrion is the primary organelle for oxygen utilization to generate bioenergy.^1^ While the cytosol and other organelles have neutral and acidic pH, the mitochondrial pH (pH_mito_) is alkaline (pH ∼ 8.0) in resting cells.^2^ Proton (H^+^) extrusion *via* the respiratory electron transport chain provides a proton motive force (*Ψ*_H^+^_), which consists of a pH gradient across the mitochondrial membrane.^3^ Mitochondria have also been shown to dynamically change in their morphology, cellular location, and distribution. They constantly undergo remodeling through fission, fusion, biogenesis, and autophagy, as well as transport to specific sites with high energy demands.^4^ The disruption of mitochondrial dynamics could be directly linked to apoptosis, cancer, aging, and neurodegenerative disorders.^5^ As such, molecular imaging of mitochondrial dynamics could provide information in the study of human diseases and drug development.^6^ However, the variation of pH_mito_ values along with their subcellular distribution, specific location, and morphological change has not been well established. Therefore, a method for quantitative imaging of pH_mito_ in living cells and tissues holds promise to elucidate mitochondria-related physiology and diseases.

To target the pH_mito_, pH-sensitive green fluorescent protein (GFP) mutants have been developed and successfully utilized to confirm the alkaline pH of the mitochondria.^7^ However, this approach suffers from variations in different cell types and technical difficulties when used with live tissues and animals.^8^ Recently, small-molecule probes for pH_mito_ based on the fluorescence off-on response have been reported.^9^ However, these probes utilized a single detection window and/or double excitation source that limit quantitative measurements due to the presence of experimental artifacts such as probe distribution, incident laser power, detection sensitivity, and photobleaching. Moreover, the p*K*_a_ values of these probes are in the acidic-neutral range (6.2–7.4), which is far from the ideal measurement of pH_mito_ (pH ∼ 8.0). In addition, live cell imaging with these probes required short excitation wavelengths that can cause photodamage and the artificial production of reactive oxygen species (ROS).^10^

Two-photon microscopy (TPM) is a fast growing domain in biomedical research, because it utilizes two near IR photons and offers higher spatial resolution with minimum background emission, deeper tissue penetration depth, and longer observation time.^11^ In combination with TPM, an emission ratiometric probe with a single excitation wavelength would be an ideal method for quantitative imaging analysis of pH_mito_. The emission ratio simultaneously reflects the population of the protonated and deprotonated forms of the fluorescent emitter, which can cancel out the aforementioned experimental artefacts. Recently, a variety of small-molecule TP probes and their utilities in bioimaging applications have been reported.^12^–^16^ However, no ratiometric TP probe for pH_mito_ have been exploited. Hence, there is a strong need to develop an emission ratiometric TP probe for pH_mito_ with a p*K*_a_ value near 8.0.

**Scheme 1:**
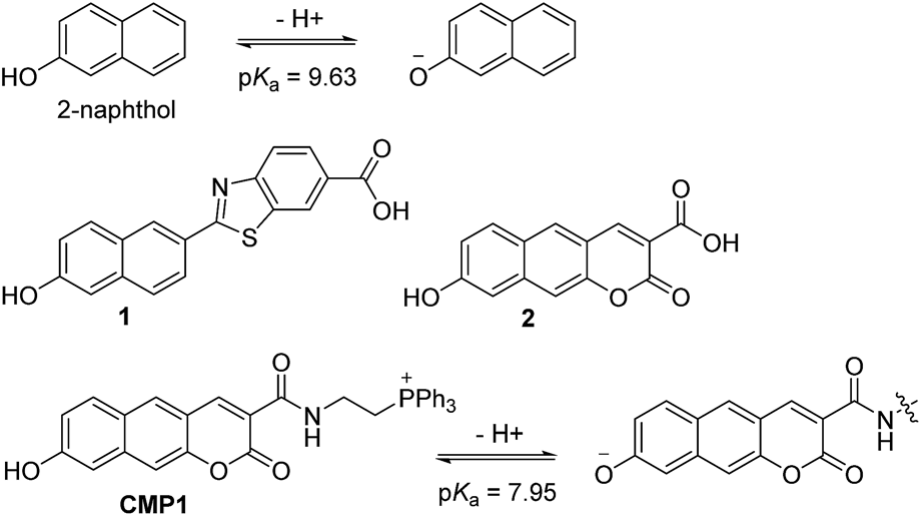
Structures of 2-naphthol, **1**, **2**, and **CMP1**, and proposed mechanism of the equilibrium on 2-naphthol and **CMP1** and their deprotonated form.

Recently, we have developed a benzimidazole-derived ratiometric TP probe (p*K*_a_ = 5.82) for acidic pH detection, which has an electron donor–acceptor substituted dipolar character.^17^ The probe showed red-shifts in its spectrum upon protonation of the benzimidazole as a weak-base, owing to the increased electron withdrawing ability and the enhanced intramolecular charge transfer (ICT). However, this approach, utilizing protonation of a weak base, makes it difficult to modify the p*K*_a_ to a value near 8.0. Thus, there is need to employ a weak acid for development of pH_mito_ sensitive probe. In this work, we report a 2-naphthol-derived ratiometric TP probe (**CMP1**, Scheme 1) for pH_mito_ that can quantitatively monitor subcellular pH_mito_ values in living cells and tissues.

## Results and discussion

We initiated this study using 2-naphthol, which has a p*K*_a_ value of 9.63 and shows a red-shifted emission maximum with pH change from acidic to basic (357 to 409 nm).^18^ The spectral red-shift under basic conditions can be attribute to an enhanced ICT process due to the stronger electron donating ability of the deprotonated form (O^–^) than the OH (*σ*^+^ = –2.3 for O^–^*vs.* –0.92 for OH) (Scheme 1).^19^ We anticipated that the p*K*_a_ value of 2-naphthol derivatives could be optimized to the mitochondrial pH by employing an electron withdrawing group and extended π-conjugated system. These translations would stabilize the conjugated base (O^–^), giving a lower p*K*_a_ value than that of 2-naphthol, along with emission ratiometric character. Therefore, we firstly prepared compound **1** containing a benzothiazolyl electron withdrawing group (Scheme 1). Benzochromene-2-one derivative (**2**) was also synthesized as a linear π-conjugating system with the expectation of more effective ICT than **1**.14*b* The synthetic route for these compounds is described in the ESI.†

**Fig. 1:**
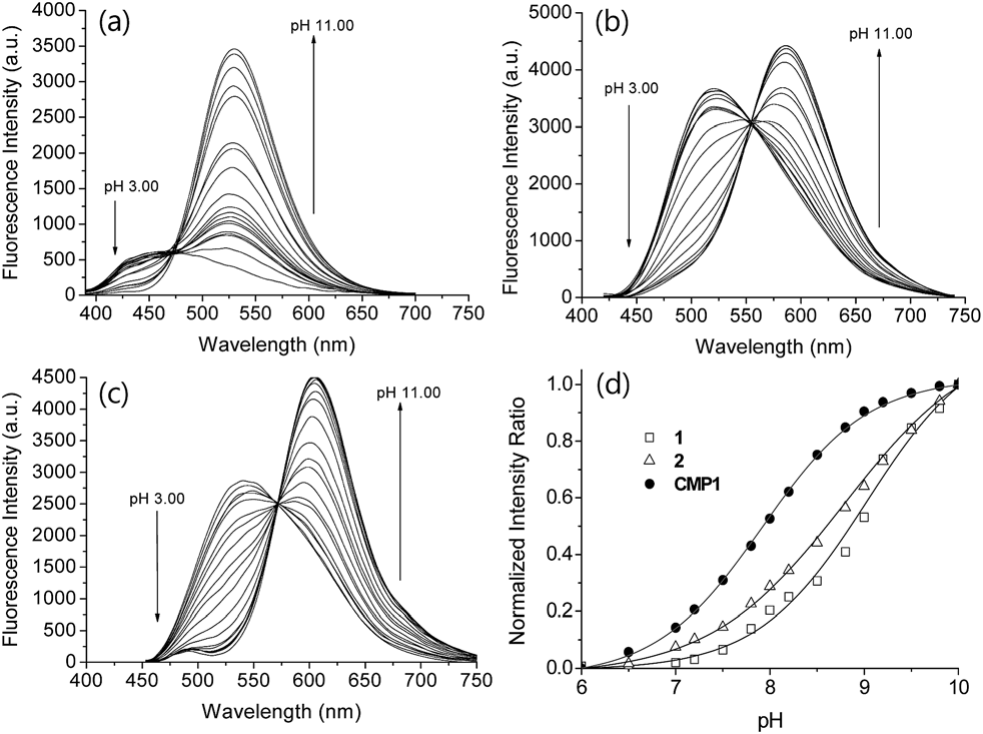
The change in fluorescence emission spectra of (a) **1**, (b) **2**, and (c) **CMP1** with various pH in buffer solution. (d) Plots of normalized intensity ratio *I*_basic_/*I*_iso_*versus* pH. The *I*_basic_ was measured at 529, 587 and 604 nm for **1**, **2**, and **CMP1**, respectively. The excitation wavelengths for **1**, **2**, and **CMP1** were 375, 400 and 425 nm, respectively.

Photophysical properties of **1** and **2** were measured in buffer solutions of different pH. Under acidic conditions (4.0), **1** displayed an absorption maximum (*λ*_abs_) at 344 nm and a fluorescence emission maximum (*λ*_fl_) at 480 nm with a large Stokes shift of 136 nm (Table 1). When the pH was changed from acidic (4.0) to basic (10.0), the *λ*_abs_ and *λ*_fl_ of **1** were shifted to the longer wavelengths at 380 nm and 530 nm, respectively, and with an increased fluorescence quantum yield (Fig. 1a and Table 1). These red-shifts might reflect the presence of the phenolate form at basic pH, as a stronger electron donor than the phenol form, thereby boosting the ICT processes. The p*K*_a_ value was calculated from the titration curve of the emission ratios (*I*_basic_/*I*_iso_) at the isoemission point (*I*_iso_) and *λ*_fl_ at pH 10.0 (*I*_basic_). The p*K*_a_ value for **1** was 9.04 ± 0.07, which is a markedly reduced value as compared to 2-naphthol. At pH 4.0, **2** showed more red-shifted *λ*_abs_ and *λ*_fl_ than those for **1**, presumably due to the enhanced ICT. As the pH of the solution was changed from acidic to basic, the intensity of fluorescence of **2** at 520 nm was gradually decreased with a simultaneous increase in intensity at 585 nm (Fig. 1b). The p*K*_a_ value of **2** is found to be 8.41 ± 0.07, lower than the value for **1**.

**Table 1:** Photophysical data for **1**, **2**, and **CMP1** in buffer solution

Probe	pH^*a*^	*λ* (1) max ^*b*^ (10^–4^*ε*)	*λ* fl max ^*c*^	*Φ* ^*d*^	p*K*_a_^*e*^	*λ* (2) max ^*f*^	*δ* ^*g*^
**1**	4.0	344 (1.14)	479	0.29	9.04 ± 0.07	740	22
	10.0	380 (1.61)	530	0.40		750	69
**2**	4.0	366 (1.25)	520	0.090	8.41 ± 0.07	750	24
	10.0	412 (1.28)	585	0.074		910	50
**CMP1**	4.0	370 (1.35)	542	0.12	7.95 ± 0.04	750	32
	10.0	453 (1.64)	604	0.084		910	62

^*a*^All the measurements were performed in buffer solution. 0.1 M KH phthalate buffer solution (pH 4.0) and 0.025 M sodium borate buffer solution (pH 10.0).

^*b*^ *λ*_max_ of the one-photon absorption spectra in nm. The numbers in parentheses are molar extinction coefficients in M^–1^ cm^–1^.

^*c*^ *λ*_max_ of the one-photon emission spectra in nm.

^*d*^Fluorescence quantum yield.

^*e*^p*K*_a_ values measured by one-photon mode.

^*f*^ *λ*_max_ of the two-photon excitation spectra in nm.

^*g*^Two-photon absorption cross-section in 10^–50^ cm^4^ s per photon (GM).

Next, we utilized **2** to prepare the mitochondria targeting probe (**CMP1**) by attaching triphenylphosphonium salt (TPP), a well-known mitochondria staining anchor,^20^ through an amide linkage (Scheme 1). Upon changing the pH from acidic to basic, the spectral shifts for **CMP1** were nearly identical to those of **2**, except that both *λ*_abs_ and *λ*_fl_ of **CMP1** and the phenolate form were shifted to the longer wavelength range (Fig. 1c, Table 1). These observations are likely due to the combined effects of the stronger electron withdrawing ability of the amide group than the carboxyl group (*σ*^–^ = 0.31 for CO_2_^–^*vs.* 0.61 for CONH_2_)^19^ and the inductive effect of the phosphonium cation. Consistently, the p*K*_a_ value of **CMP1** is 7.95 ± 0.05, a lower value than for **2** and well-matched to mitochondrial pH (8.0). Further, the plot of the emission ratio with pH indicated that this probe would be suitable for detecting over the pH range of 6.0–9.0 (Fig. 1d). The ratio (*I*_604_/*I*_540_) of **CMP1** was robustly cycled back and forth in the pH changes between pH 5.0 and 9.0 (Fig. S8, ESI†), indicating the reversible sensing ability. In addition, the ratio of **CMP1** in buffer solutions was unperturbed in the presence of biologically relevant metal ions, thiol species, ROS, reactive nitrogen species, and hydrogen sulfide (Fig. S9, ESI†). Consequently, **CMP1** showed a marked yellow-to-red emission color change in response to a change in pH from acidic to basic and an appropriate p*K*_a_ value for assessing the change of pH_mito_ without interference from other physiological species.

**Fig. 2:**
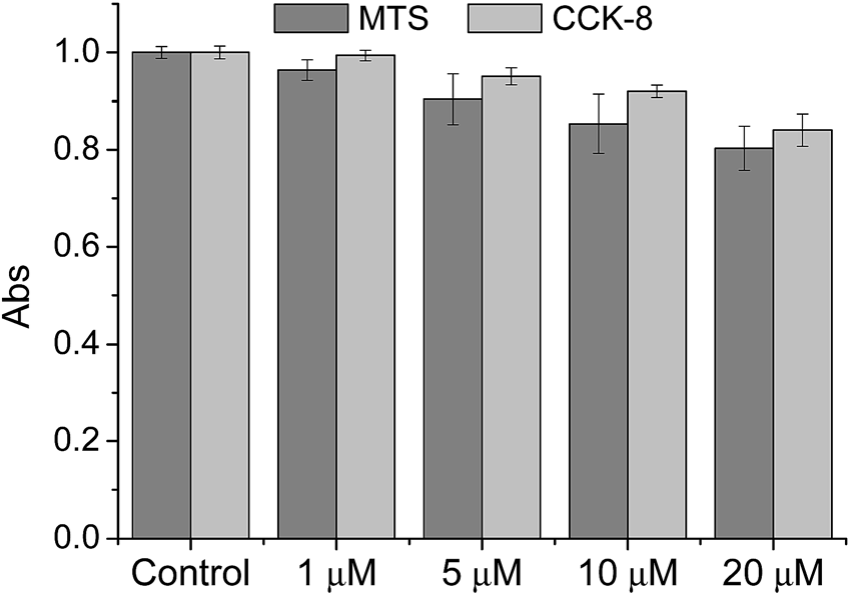
Viability of HeLa cells in the presence of **CMP1** as measured by using MTS and CCK-8 assays. The cells were incubated with 0–20 μM **CMP1** for 2 h.

We then tested the ability of **CMP1** as a TPM imaging probe. The maximum two-photon absorption cross section (*δ*_max_) values of **1**, **2**, and **CMP1** at pH 4.0 and 10.0 were in the range of 20–70 GM, which are comparable to the values of existing small-molecule TP probes (Table 1 and Fig. S10, ESI†).12*b* Further, the TPM images of the **CMP1**-labeled HeLa cells and primary cultured astrocytes showed bright tubule-like networks, which might be the mitochondria. This probe displayed high photostability under the imaging conditions (Fig. S11, ESI†) and low cytotoxicity as determined by MTS and CCK-8 assays (Fig. 2).

**Fig. 3:**
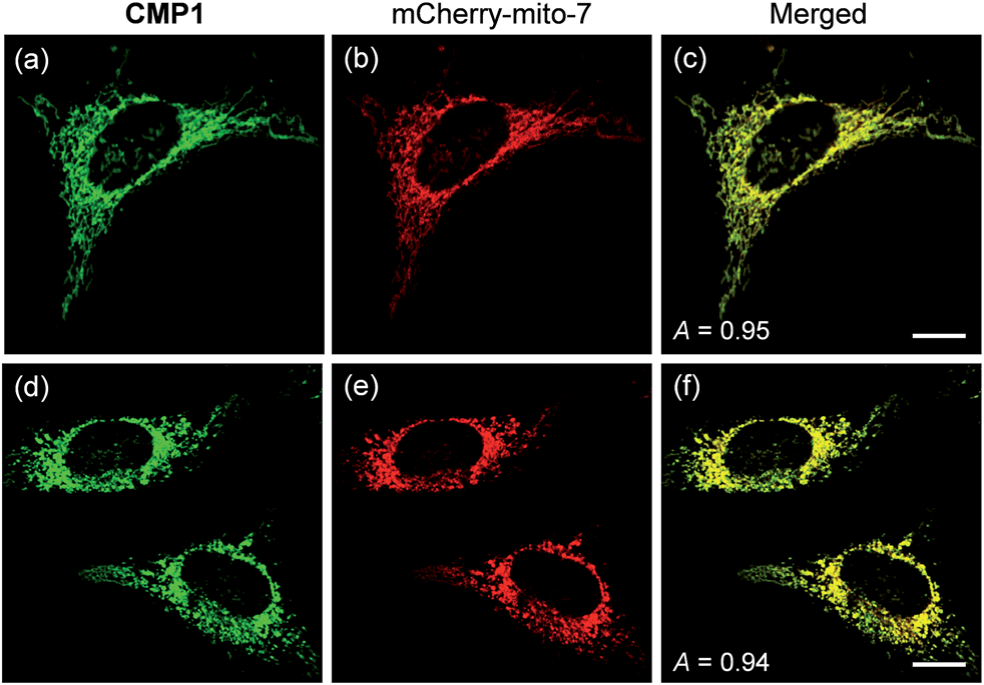
(a and d) TPM and (b and e) OPM images of co-labeled with 2 μM **CMP1** and mCherry-mito-7, (a–c) before and (d–f) after pretreatment with 20 μM CCCP for 2 h. (c and f) Merged image. The wavelengths for OP and TP excitation were 552 and 820 nm respectively, and the corresponding emission were collected at 450–550 nm (**CMP1**) and 650–700 nm (mCherry-mito-7). Scale bars: (c) 10 and (f) 16 μm.

To demonstrate the mitochondrial staining ability of the probe, we conducted co-localization experiments with mCherry-mito-7, a well-known red-emissive protein for mitochondria.^21^ The TPM image of **CMP1** merged well with the mCherry-mito-7 image (Fig. 3a–c). The Pearson's co-localization coefficient (*A*) was 0.95 ± 0.05, which indicated that **CMP1** predominantly resided in the mitochondria. A similar result was also obtained when the co-localization experiment was performed with the molecular marker, MitoTracker Red (*A* = 0.88 ± 0.05) (Fig. S12, ESI†). Upon treatment of CCCP (carbonyl cyanide *m*-chlorophenyl hydrazone), a protonophore that promotes the release of intra-mitochondrial cations and causes rapid acidification by collapsing the mitochondrial membrane potential (Δ*Ψ*_m_),^22^ the *A* values between the images of **CMP1** and mCherry-mito-7 were found to be 0.94 ± 0.05 (Fig. 3d–f). Because it has been well-established that the staining ability of mCherry-mito-7 does not depend on the Δ*Ψ*_m_,^21^ this result confirmed the robust staining ability of **CMP1** for the mitochondria regardless of the alteration of Δ*Ψ*_m_ (Fig. 3).

**Fig. 4:**
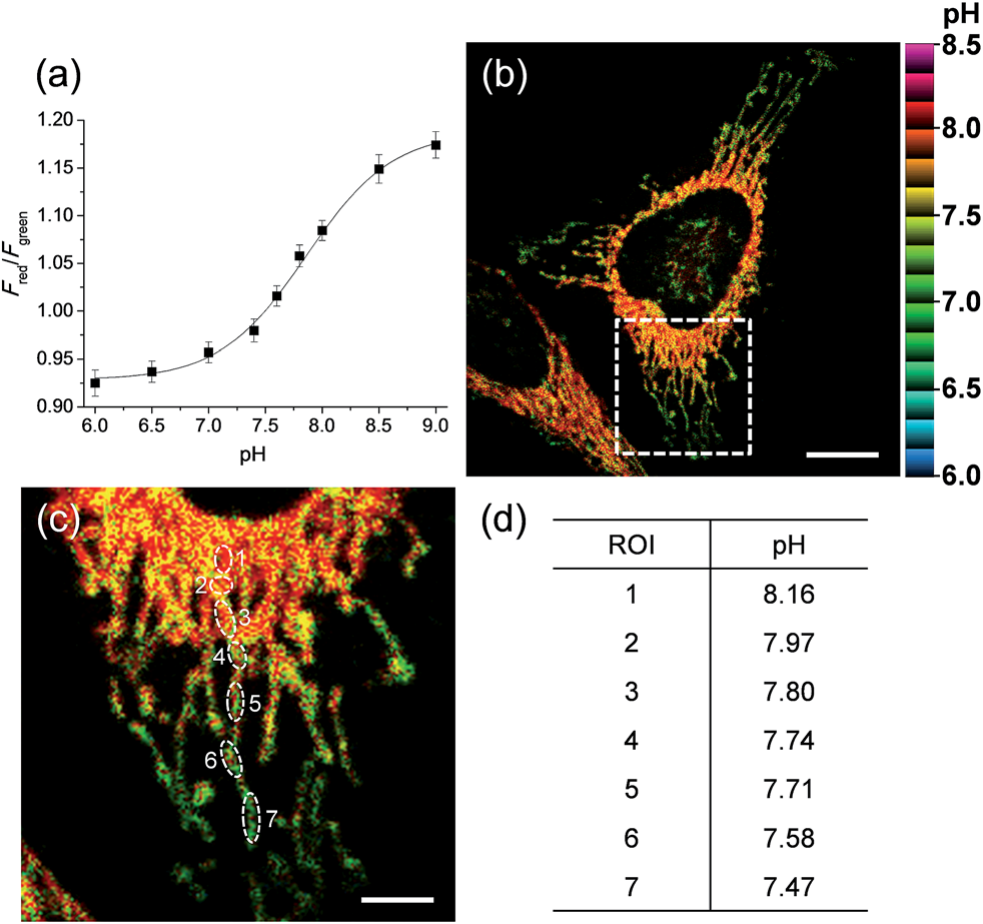
(a) Two-photon *F*_red_/*F*_green_ titration with various pH in ionophore-treated and **CMP1**-labeled HeLa cells. (b) Pseudocolored ratiometric TPM images (*F*_red_/*F*_green_) of HeLa cells stained with 2 μM **CMP1**. (c) Higher magnification of white boxed area in (b). (d) The estimated pH values at the region of interest (ROI) indicated by white rounds in (c). Excitation wavelength: 820 nm. Scale bars: (b) 12 and (c) 4 μm.

We next tested the probe to determine the pH_mito_ value in live cells using TPM. Live cell imaging was carried out at 37 °C using an incubating chamber for maintaining humidity and pH. To find the optimal detection window for ratiometric imaging, the TP excited fluorescence (TPEF) spectra were collected from ionophore-treated HeLa cells labeled with **CMP1** at pH 4.0 and 10.0. Upon excitation at 820 nm, the TPEF spectra at pH 4.0 and pH 10.0 were similar to those measured in buffer solution, in which the *λ*_fl_ maxima (505 and 569 nm at pH 4.0 and 10.0) are slightly blue-shifted from those measured in buffer solution (Fig. S13, ESI†). These results indicated that the polarity of the probe environment within the cells was rather homogeneous and slightly more hydrophobic than in the buffer solution.^17^ This consequence allowed ratiometric imaging (*F*_red_/*F*_green_) for pH_mito_ by using 450–500 nm (*F*_green_) and 550–600 nm (*F*_red_) as most sensitive windows. The pH calibration curve was obtained by using *F*_red_/*F*_green_ of ionophore-treated and **CMP1**-labeled HeLa cells (Fig. 4a). The p*K*_a_ of **CMP1** in cells was 7.86 ± 0.05, a nearly identical value as that measured in buffer solution. In addition, the plots of *F*_red_/*F*_green_*vs.* pH value indicated that **CMP1** is suitable for measuring the pH_mito_ in the range of pH 6.0–9.0.

More importantly, the ratiometric TPM image (*F*_red_/*F*_green_) of **CMP1**-labeled HeLa cells clearly displayed various pH_mito_ values ranging from 7.4 to 8.2, indicating the heterogeneity of pH_mito_ (Fig. 4b and c). In addition, there was a denser population of mitochondria around the nucleus than in the periphery. Interestingly, Fig. 4b–d shows that the mitochondria in the perinuclear position had higher pH values in comparison to mitochondria at the periphery of cells. This difference might reflect a mitochondrial region-specific function.^23^ It has been well established that perinuclear mitochondria are mainly involved in mitochondria-metabolized ATP generation by regulating nuclear function by the assimilated phosphotransfer network between mitochondria and the nucleus.23*b* The higher pH values in the perinuclear region may be linked to the local metabolic state, thus indicating to the need for future studies to elucidate its physiological function. Similar results were observed with primary cultured astrocytes (Fig. 7).

**Fig. 5:**
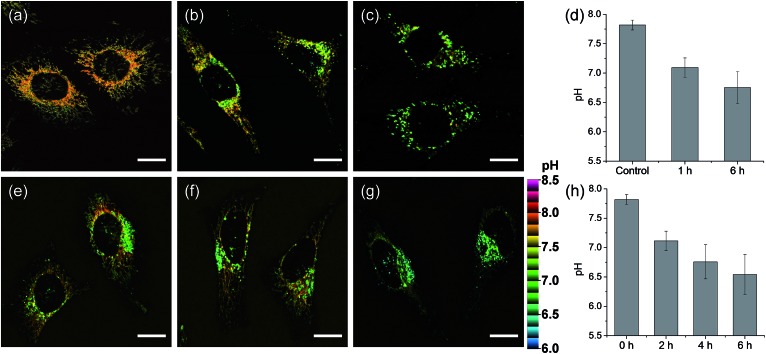
Pseudocolored ratiometric TPM images (*F*_red_/*F*_green_) of HeLa cells stained with 2 μM **CMP1**. (a) Control image. (b and c) After pretreated with 20 μM CCCP for (b) 1 h and (c) 6 h. (e–g) The cells were starved in amino acids/serum-deprived buffer solution for (e) 2 h, (f) 4 h and (g) 6 h to induce autophagy. (d and h) The average and standard deviation of the pH values that were estimated from the *F*_red_/*F*_green_ ratios in the TPM images. Excitation wavelength: 820 nm. Scale bars: 15 μm.

We next monitored the change of pH_mito_ with carbonyl cyanide *m*-chlorophenyl hydrazone (CCCP) treatments and under starvation conditions. The average value of pH_mito_ of **CMP1**-labeled HeLa cells was 7.82 ± 0.08. Upon treatment with CCCP for 1 h and 6 h, the average values of pH_mito_ decreased to 7.09 ± 0.16 and 6.75 ± 0.27, respectively (Fig. 5a–d). In addition, the ratio TPM imaging clearly displayed that the perinuclear mitochondria preferentially adopted a globular morphology with acidic pH, compared to the peripheral mitochondria (Fig. 5b and c) and the process extended to the periphery. Moreover, higher magnification images clearly showed the pH values of globular structures ranged from 7.40 to 6.36 (Fig. 5c), which were significantly decreased values by 0.7–1.8 pH units as compared to those for long and highly interconnected structures. Similar results were detected in the cell starvation process. It is well-established that the damaged mitochondria can be eliminated by cell degradation processes, *i.e.*, autophagy, a process in which mitochondria that have attained an acidic pH are entrapped in acidic lysosomes followed by hydrolytic digestion.^24^ Upon nutrient deprivation for 2–6 h (Fig. 5e–h), a globular morphology was noted in mitochondria near the nucleus appeared and this population of altered mitochondria increased with time; during this time, the pH values ranged from 7.1 to 6.2 (Fig. 5e–g).

**Fig. 6:**
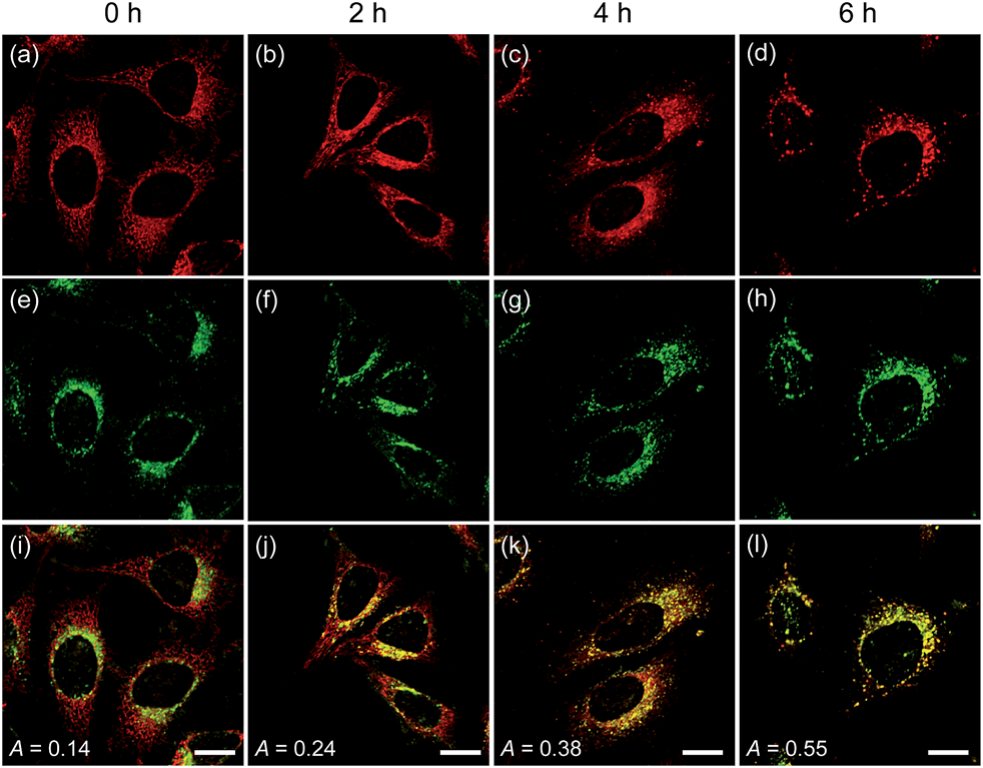
TPM images of HeLa cells co-labeled with (a–d) **CMP1** and (e–h) BLT-blue. (i–l) Merged images. The cells were starved in an amino acid/serum-deprived buffer solution for 0–6 h to induce autophagy. The excitation wavelength were 750 nm (BLT-blue) and 820 nm (**CMP1**), and corresponding emission were collected at 400–450 (BLT-blue) and 550–600 nm (**CMP1**), respectively. Scale bars: 20 μm.

To confirm these processes, HeLa cells were co-labeled with **CMP1** and BLT-blue,^25^ a TP LysoTracker that emits separate TPEF from **CMP1** in the cells (Fig. S14, ESI†). The dual-color TPM images (Fig. 6) clearly exhibited the autophagy processes. The Pearson's co-localization coefficient (*A*) was found to be 0.14 ± 0.5 before stimulating the cells (Fig. 6a, e and i). When the cells were starved in nutrient-deprived buffer solution, the *A* value increased to 0.24 to 0.38 to 0.55 after starvation for 2 h, 4 h and 6 h, respectively (Fig. 6). Hence, these outcomes clearly demonstrated that **CMP1** is capable of monitoring the change of pH_mito_ values in live cells while defining the dynamic shapes of mitochondria at specific positions.

**Fig. 7:**
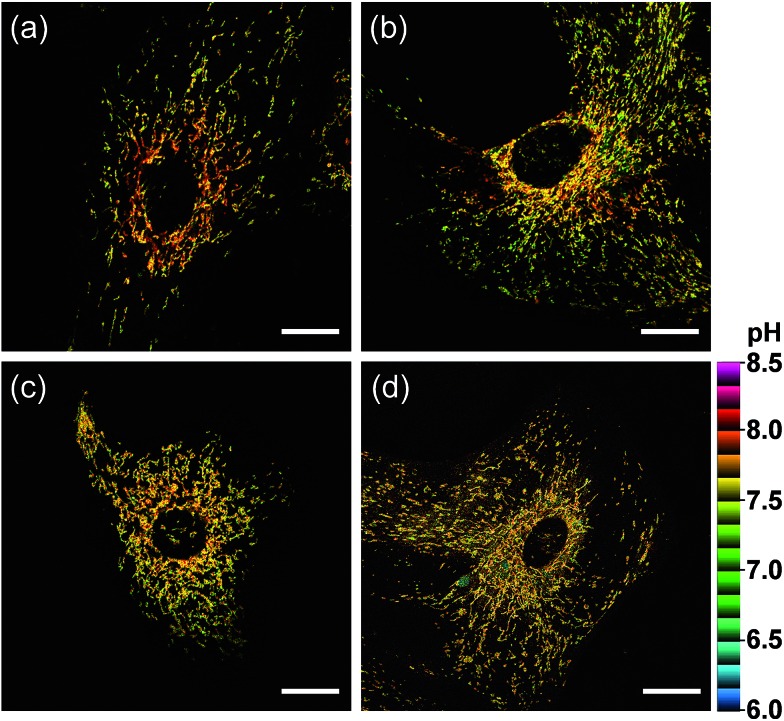
Pseudocolored ratiometric TPM images (*F*_red_/*F*_green_) of astrocytes extracted from (a) wild type, (b) DJ-1-knockout (KO), (c) Parkin-KO and (d) Pink-KO mice brains were stained with 2 μM **CMP1**. Excitation wavelength: 820 nm. Scale bars: 20 μm.

We further utilized **CMP1** to measure the pH_mito_ values in Parkinson's disease (PD) model astrocytes such as DJ-1, Parkin, and Pink gene knockout (KO) astrocytes. These astrocytes contain a PD-related gene that has multiple functions. The ratiometric TPM imaging with **CMP1** exhibited the average pH_mito_ value of 7.78 ± 0.09 in wild-type (WT) astrocytes (Fig. 7a). Moreover, the distribution of pH_mito_ were similar between PD models and WT astrocytes, with the exception that the average pH_mito_ values of PD models are slightly lower (*ca.* 0.1–0.2 pH unit) than those for WT astrocytes (Fig. 7b–d). This feature suggests the potential use of **CMP1** in clinical applications.

**Fig. 8:**
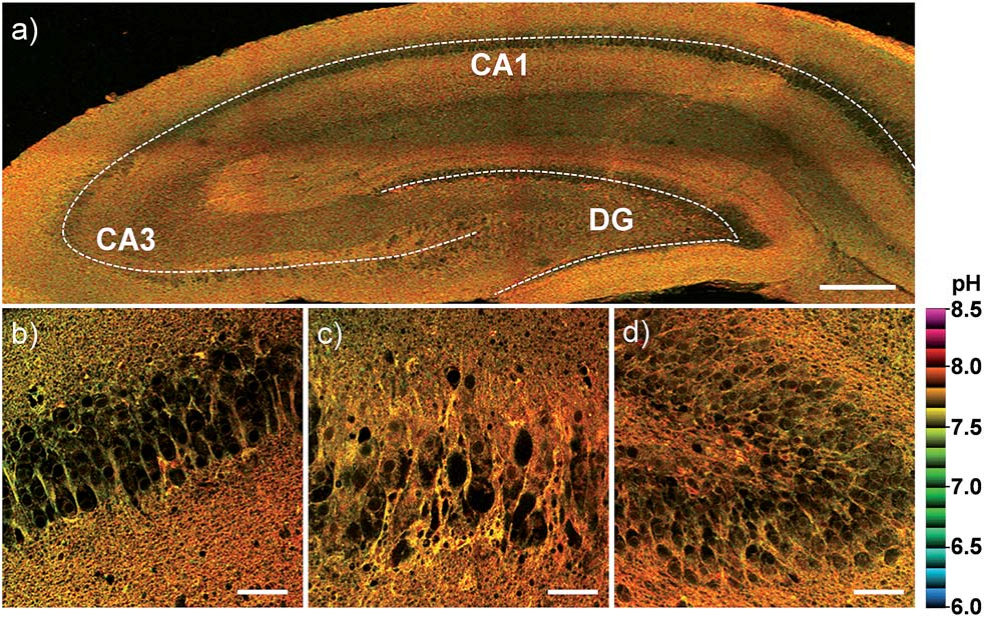
Pseudocolored ratiometric TPM images (*F*_red_/*F*_green_) of a rat hippocampal slice stained with 20 μM **CMP1**. (a) 360 TPM images along the *z*-direction at the depths of approximately 90–210 μm were accumulated to visualize the overall pH_mito_ distribution with 10× magnification. (b–d) Higher magnification images (40×) at the regions of (b) CA1, (c) CA3 and (d) DG. Excitation wavelength: 820 nm. Scale bars: (a) 300 and (b–d) 48 μm.

Finally, we investigated the utility of **CMP1** in live tissue imaging. The ratiometric TPM images were obtained from a fresh slice of rat hippocampus, a region of the brain that is important for learning and memory. The accumulated 360 TPM images collected at 90–210 μm depths provided the overall pH_mito_ distribution through the regions of CA1, CA3, and DG (dentate gyrus) (Fig. 8). They showed the average pH_mito_ values of these regions in 7.86–7.88 (Fig. 8), in which higher pH values of approximately 8.2 (red spots) are mainly appeared in the DG (Fig. 8a and d). Moreover, the higher magnification images at these regions revealed the heterogeneous pH_mito_ distribution at the tissue level.

## Conclusions

We have developed a ratiometric two-photon probe (**CMP1**) for quantitative measurement of pH_mito_ in live cells and tissues. This probe, designed with the aim of controlling ICT in 2-naphthol, has a p*K*_a_ value of 7.86 ± 0.05 in the cells, and shows a marked yellow to red emission color change in response to pH alterations from 6.0 to 9.0. **CMP1** exhibits easy cell loading, robust staining ability of mitochondria, low cytotoxicity, and bright TPEF *in situ*, thereby allowing quantitative detection of the pH_mito_ in live cells and tissues. The ratiometric TPM imaging studies by using **CMP1** clearly reveal the heterogeneity of pH_mito_ values with respect to the specific location of mitochondria within the cells, along with their different shapes. Further, we found that the pH_mito_ values of mitochondria in the perinuclear region are higher than those at the periphery of cells. The average pH_mito_ values of PD model astrocytes are slightly more acidic than those for WT astrocytes. These findings demonstrate that **CMP1** will be useful as a quantitative imaging probe to study pH_mito_ in biomedical research.

## Experimental sections

### Spectroscopic measurements

Absorption spectra were recorded on S-3100 UV-Vis spectrophotometer and fluorescence spectra were obtained with FluoroMate FS-2 fluorescence spectrophotometer with a 1 cm standard quartz cell. The fluorescence quantum yield was determined by using Coumarin 307 (*Φ* = 0.95 in MeOH) and Rhodamine 6G (*Φ* = 0.95 in MeOH) as the reference by the literature method.^26^

### p*K*_a_ value

Fluorescence pH titrations were performed in buffer solution [pH 3.0, pH 4.0, pH 5.0 (0.1 M KH phthalate buffer solution), pH 6.0, pH 6.5, pH 7.0, pH 7.2, pH 7.5, pH 7.8, pH 8.0, pH 8.2, pH 8.5 and pH 8.8 (0.1 M KH_2_PO_4_ buffer solution), pH 9.0, pH 9.2, pH 9.5, pH 9.8 and pH 10.0 (0.025 M sodium borate buffer solution), pH 11.0 (0.05 M NaHCO_3_ buffer solution)]. For **1**, **2** and **CMP1**, a 3.0 μL of the stock solution of probe in DMSO (1 × 10^–3^ M) was added to cuvette containing 3.0 mL of buffer solution to prepared 1.0 μM of probe solution and the spectral change in the fluorescence were measured as a function of pH (3.0–11.0), p*K*_a_ values of **1**, **2** and **CMP1** were calculated by linear regression analysis fluorescence data to fit the following equation.^27^
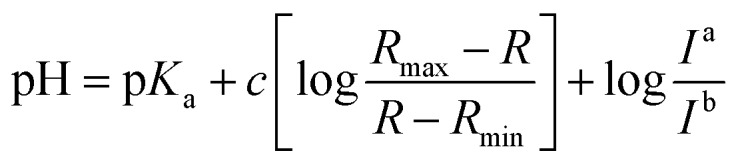
where *R* is the observed ratios (*I*_529_/*I*_iso_ for **1**, *I*_587_/*I*_iso_ for **2** and *I*_604_/*I*_iso_ for **CMP1**, respectively) at given pH. *R*_max_ and *R*_min_ are the maximum and minimum limiting values of *R* and *c* is the slope. *I*^a^/*I*^b^ is the ratio of the fluorescence intensity in acid (pH 3.0) to the intensity in the base (pH 11.0) at the wavelength chosen for the denominator of *R*. In these cases, this correction vanishes by using the sharp isoemissive point (472 nm for **1**, 554 nm for **2** and 572 nm for **CMP1**).

### Measurement of two-photon cross section

The two-photon cross section (*δ*) was determined by using femto second (fs) fluorescence measurement technique as described.^28^**1**, **2** and **CMP1** (1.0 × 10^–6^ M) were dissolved in buffer solution (pH 4.0 and 10.0) and the two-photon induced fluorescence intensity was measured at 720–950 nm by using Rhodamine 6G as the reference, whose two-photon property has been well characterized in the literature.^29^ The intensities of the two-photon induced fluorescence spectra of the reference and sample emitted at the same excitation wavelength were determined. The TPA cross section was calculated by using following equation
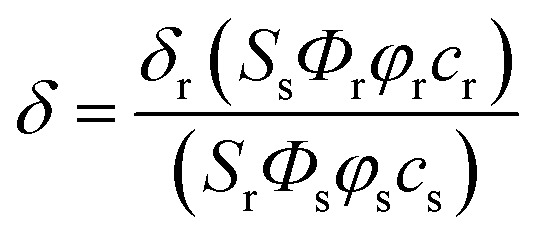
where the subscripts s and r stand for the sample and reference molecules. The intensity of the signal collected by a CCD detector was denoted as *S*. *Φ* is the fluorescence quantum yield. *φ* is the overall fluorescence collection efficiency of the experimental apparatus. The number density of the molecules in solution was denoted as *c*. *δ*_r_ is the TPA cross section of the reference molecule. Fig. S9, ESI† represents the two-photon spectra of **1**, **2** and **CMP1** in different buffer solution conditions.

### Cell culture

All the cells were passed and plated on glass-bottomed dishes (NEST) before imaging for two days. They were maintained in a humidified atmosphere of 5/95 (v/v) of CO_2_/air at 37 °C. The cells were treated and incubated with 2 μM **CMP1** at 37 °C under 5% CO_2_ for 30 min, washed three times with phosphate buffered saline solution (PBS; Gibco), and then imaged after further incubation in colorless serum-free media for 30 min. The culture mediums for each cells are as below.

HeLa human cervical carcinoma cells (ATCC, Manassas, VA, USA): MEM (WelGene Inc, Seoul, Korea) supplemented with 10% FBS (WelGene), penicillin (100 units per mL), and streptomycin (100 μg mL^–1^). Primary astrocytes were cultured from the cortex of DJ-1-KO, Parkin-KO or WT mice brains. In brief, cortexes were removed and triturated in DMEM (Invitrogen, Carlsbad, CA, USA) containing 10% FBS (HyClone, Logan, UT, USA), plated in 75 cm^2^ T-flasks (0.5 hemisphere per flask), and incubated for 2–3 weeks. Microglia were detached from flasks by mild shaking, filtered through a nylon mesh to remove cell clumps, and cultured in DMEM containing 10% FBS.^30^ Astrocytes remaining in the flask were harvested with 0.1% trypsin and cultured in DMEM containing 10% FBS.

### Two-photon fluorescence microscopy

Two-photon fluorescence microscopy images of **CMP1** labeled cells and tissues were obtained with spectral confocal and multiphoton microscopes (Leica TCS SP8 MP) with ×10 dry, ×40 oil and ×100 oil objectives, numerical aperture (NA) = 0.30, 1.30, and 1.30. The two-photon fluorescence microscopy images were obtained with a DMI6000B Microscope (Leica) by exciting the probes with a mode-locked titanium-sapphire laser source (Mai Tai HP; Spectra Physics, 80 MHz, 100 fs) set at wavelength 820 nm and output power 2901 mW, which corresponded to approximately 6.26 × 10^8^ mW cm^–2^ average power in the focal plane. Live cell imaging was performed using the live cell incubator systems (Chamlide IC; Live Cell Instrument) for stable cell environment by maintaining proper temperature, humidity and pH over the long term. To obtain images at 450–500 nm (*F*_green_) and 550–600 nm (*F*_red_) range (Fig. S13a, ESI†) internal PMTs were used to collect the signals in an 8 bit unsigned 512 × 512 and 1024 × 1024 pixels at 400 and 200 Hz scan speed, respectively. Ratiometric image processing and analysis was carried out using MetaMorph software.

### Cell viability

To evaluate the cytotoxic effect of **CMP1** in HeLa cells, MTS (cell Titer 96H; Promega, Madison, WI, USA) and CCK-8 kit (Cell Counting Kit-8; Dojindo, Japan) assay were performed according to the manufacture's protocol. The results are shown in Fig. 2, which revealed that the **CMP1** has low cytotoxicity at its different concentration in our incubation condition.

### Co-localization experiments

Co-localization experiments were conducted by co-staining the HeLa cells and astrocytes with appropriate combinations of **CMP1** (2 μM), MTR (1 μM), mCherry-mito-7 and BLT-blue (0.5 μM) for 30 min. TPM and OPM images were obtained by collecting the emissions at 400–450 (BLT-blue), 450–500 and 550–600 (**CMP1**) and 650–700 (MTR, mCherry-mito-7), respectively. The two different detection windows for **CMP1** were adopted upon requirement for colocalization experiments. The background images were corrected, and the distribution of pixels in the OPM and TPM images acquired in the green and red channels, respectively, was compared by using scatter gram. The Pearson's co-localization coefficients (*A*) were calculated by using AutoQuant X2 program.

### Cell calibration

A pH calibration curve was generated by *F*_red_/*F*_green_ of ionophores-treated and **CMP1**-labeled HeLa cells. The cells were treated and incubated with 2 μL of **CMP1** in DMSO sock solution (1.0 mM) at 37 °C under 5% CO_2_ for 30 min, and then the extracellular media was replaced with 1 mL of calibration buffer (125 mM KCl, 20 mM NaCl, 0.5 mM CaCl_2_, 0.5 mM MgCl_2_, 5 μM nigericine, 5 μM monensin, and 25 mM buffer; MES for pH 6.0 and 6.5, HEPES for pH 6.5–8.0 and Tris for pH 8.5–9.0).7*a* The cells were treated with the calibration buffer for 15–20 min at room temperature. The TPEF intensity at 450–500 nm (*F*_green_) and 550–600 nm (*F*_red_) of **CMP1** (Fig. S13a, ESI†) well changed with pH, and we obtained pH calibration curve the plots of *F*_red_/*F*_green_*versus* pH values.

### Preparation and staining of fresh rat hippocampal slices

Rat Hippocampal slice were prepared from the hippocampi of 2 weeks-old rat (SD) according to an approved institutional review board protocol. Coronal slices were cut into 400 μM-thicks using a vibrating-blade microtome in artificial cerebrospinal fluid (ACSF; 138.6 mM NaCl, 3.5 mM KCl, 21 mM NaHCO_3_, 0.6 mM NaH_2_PO_4_, 9.9 mM d-glucose, 1 mM CaCl_2_, and 3 mM MgCl_2_). Slices were incubated with 20 μM **CMP1** in ACSF bubbled with 95% O_2_ and 5% CO_2_ for 1 h at 37 °C. Slices were then washed three times with ACSF and transferred to glass-bottomed dishes (NEST) and observed in a spectral confocal multiphoton microscope. The TPM images obtained at about 90–210 μm depth.

## Supplementary Material

Supplementary information
