# Supplementary material for: A ratiometric two-photon probe for quantitative imaging of mitochondrial pH values
Source: Chem Sci. 2015 Oct 27;7(1):766–73. doi: 10.1039/c5sc03708e (PMC5953010; doi:10.1039/c5sc03708e)
Supplement: Supplementary file 1 [file SC-007-C5SC03708E-s001.pdf]

Electronic Supplementary Material (ESI) for Chemical Science.
This journal is © The Royal Society of Chemistry 2015

Supporting Information to Accompany "**A Ratiometric Two-Photon Probe for Quantitative Imaging of Mitochondrial pH Values**"

Avik Ranjan Sarkar‡, Cheol Ho Heo‡, Lei Xu, Hyo Won Lee, Ho Young Si, Ji Won Byun, and Hwan Myung Kim*

Department of Chemistry and Department of Energy Systems Research, Ajou University,

Suwon 443-749, Korea.

Table of Contents

	Page
**Synthesis of 1, 2** and **CMP1**.	S3
**Figure S1**. ^{1}H-NMR spectrum (400 MHz) of **1** in d_6-DMSO.	S8
**Figure S2**. ^{13}C-NMR spectrum (400 MHz) of **1** in d_6-DMSO.	S8
**Figure S3.** ^{1}H-NMR spectrum (400 MHz) of **2** in d_6-DMSO.	S9
**Figure S4**. ^{13}C-NMR spectrum (400 MHz) of **2** in d_6-DMSO.	S9
**Figure S5.** ^{1}H-NMR spectrum (400 MHz) of **CMP1** in d_6-DMSO.	S10
**Figure S6**. ^{13}C-NMR spectrum (400 MHz) of **CMP1** in d_6-DMSO.	S10
**Figure S7.** HRMS spectrum of **CMP1**.	S11
**Figure S8**. (a) The change in fluorescence emission spectra of **CMP1** with alteration of pH in buffer (0.1 M KH_2PO_4 buffer). (b) Change in fluorescence intensity ratio I_{604}/I_{540} of **CMP1** (1 μM) determined upon accomplished successive pH cycles. The excitation wavelength was 425 nm.	S12
**Figure S9.** Fluorescence intensity ratio of 1 μM **CMP1** towards different metal ions and biological redox species. 1, probe itself; 100 μM of 2, Na^+; 3, K^+; 4, Ca^{2+}; 5, Zn^{2+}; 6, Mn^{2+}; 7, Mg^{2+}; 8, Cu^{2+}; 9, Fe^{2+}; 5 mM of 10, GSH; 11, Cys; 12, Hcy; 100 μM of 13, H_2O_2; 14, O_2^-; 15, $^{\cdot}OH$; 16, $NO^{\cdot}$; 17, ClO^- ; 18, Na_2S. Bars represent the ratio (I_{604}/I_{572} for **CMP1**) of the emission intensities before and after addition of the metal ions and redox species. Data were acquired in KH_2PO_4 buffer (10 mM, pH 7.4). The excitation wavelength was 425 nm for **CMP1**.	S13
**Figure S10.** Two-photon absorption cross section spectra of (a) **1**, (b) **2** and (c) **CMP1** in buffer solution at pH 4.0 and pH 10.0, respectively.	S14
**Figure S11** (a) TPM images of **CMP1**-labeled HeLa cells. (b) The relative two-photon excited fluorescence (TPEF) intensity from the A and B of image as function of time. The digitized intensity was recorded with 2.00 sec intervals for the duration of one hour using *xyt* mode. The TPEF intensities were collected 400-650 nm 820 nm for **CMP1** with femto-second pulses. The images shown are representative of the images obtained in the repeat experiments (n = 3). Scale bar: 33 μm.	S15

Figure S12. (a,d) TPM and (b,e) OPM images of HeLa cells and astrocyte co-labeled with 2 μM **CMP1** and 1 μM MitoTracker Red FM (MTR). (c,f) Merged image from TPM image of **CMP1** and OPM image of MTR. Excitation wavelength for TPM and OPM are 820 and 552 nm; the emissions were collected at 450-550 nm (**CMP1**) and 650-700 nm (MTR). The images shown are representative of the images obtained in repeat experiments (n = 5). Scale bars: 20 μm. S16

Figure S13. (a) Two-photon excited fluorescence spectra of ionophores-treated HeLa cells labeled with 2 μM **CMP1** at pH 4.0 and pH 10.0. The excitation wavelength was 820 nm. (b) Emission spectra of **CMP1** in buffer solution at pH 4.0 and pH 10.0 and the two-photon excited fluorescence spectra to normalized emission spectra at pH 4.0 (■) and pH 10 (○) acquired from HeLa cells, respectively. S17

Figure S14. (a) Chemical structure of **BLT-blue**. (b) Normalized two-photon excited fluorescence spectra of **BLT-blue** (■) and **CMP1** (○) acquired from HeLa cells, respectively. The excitation wavelengths were 750 nm and 820 nm for **BLT-blue** and **CMP1**, respectively. S17

Synthesis of 1, 2 and CMP1. Compounds **A**[1] and **B**[2] were prepared by the literature methods and the synthesis of **1**, **2** and **CMP1** were accomplished according to following scheme S1. The synthetic procedures of intermediates, **2** and **CMP1** are described below.

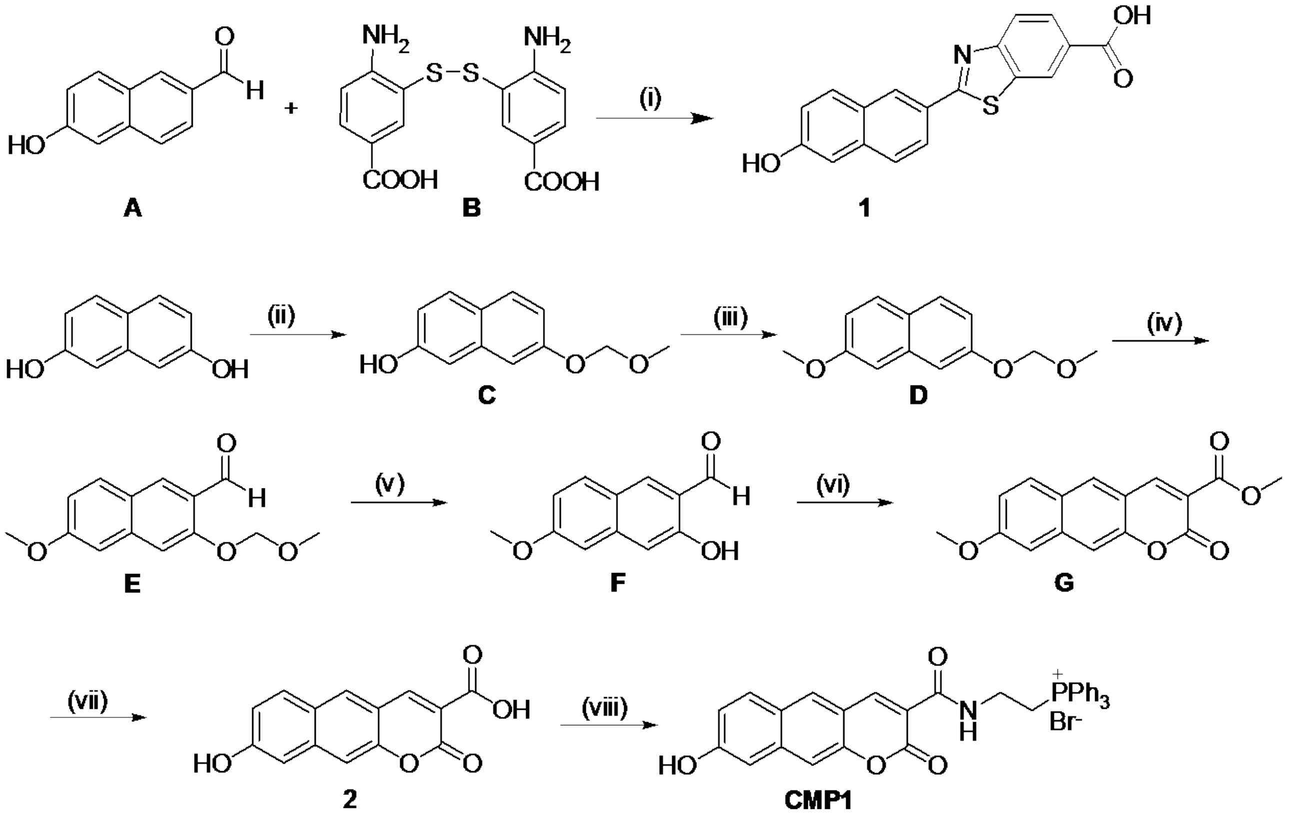

Scheme S1. Reagents and conditions: (i) *p*-TSA, DMF, 120 ^{0}C; (ii) Chloromethyl methyl ether, sodium hydride (NaH), *N,N*-Dimethylformamide (DMF), 0 ^{0}C to rt, 2hr; (iii) Iodomethane, potassium carbonate (K_2CO_3), DMF, rt, 6hrs; (iv) *tert*-Butyllithium, DMF, diethyl ether, -20 ^{0}C; (v) Isopropyl alcohol, 5(M) HCl, 65 ^{0}C, 2hr; (vi) Dimethyl malonate, piperidine, ethanol, 80 ^{0}C, 12 hrs; (vii) 48% HBr, 120 ^{0}C, 12hrs.; (viii) (2-aminoethyl)triphenylphosphonium bromide, (Benzotriazole-1-yioxy) tripyrrolidino phosphonium hexafluorophosphate (PyBOP), 1-hydroxybenzotriazole (HOBt), *N, N*-Diisopropyl ethylamine (DIPEA) , *N,N*-Dimethylformamide (DMF), rt.

Compound 1. Compound **A** (0.20 g, 1.2 mmol), **B** (0.38 g, 1.2 mmol) and *p*-toluenesulfonic acid monohydrate (0.02 g, 0.12 mmol) were dissolved in DMF (10 mL) and the reaction mixture was stirred at 120 °C under nitrogen atmosphere for 12 h. After cooling to room temperature, distilled water was added. The product was collected by filtration, washed with water, and purified by crystallization from ethanol to get the compound **2** as a yellow solid. Yield: 0.195 g (53 %); m.p. 260 °C; ^{1}H NMR (400 MHz, d_6-DMSO): δ (ppm) 13.07 (s, 1H), 10.24 (s, 1H), 8.75 (s, 1H), 8.58 (s, 1H), 8.12-8.04 (m, 3H), 8.02 (d, *J* = 8.4 Hz, 1H), 7.84 (d, *J* = 8.8 Hz, 1H), 7.20-7.17 (m, 2H). 13C NMR (100 MHz, d_6-DMSO,): δ (ppm) 171.0, 166.7, 157.2, 156.2, 136.3, 134.3, 130.7, 127.7, 127.4, 127.3, 127.1, 127.0, 126.8, 124.1, 124.0, 122.1, 119.7, 108.9.

Compound C. Commercially available naphthalene-2,7-diol (10.0 g, 62.43 mmol) was dissolved in anhydrous DMF (100 mL) and cooled under nitrogen to 0 ^{0}C, and sodium hydride (1.5 g, 62.43 mmol) was added portion wised followed by chloromethyl methyl ether (4.74 mL, 62.43 mmol). The reaction mixture was allowed to stirring at same temperature for 30 min. The reaction mixture was quenched by addition of saturated ammonium chloride solution (100 mL) and ethyl acetate was added thereto and the whole reaction mass was allowed stirring for 30 min. Collect the organic layer washed with water (2 x 50 mL), dried over anhydrous Na_2SO_4 and concentrated under reduced pressure to obtained the crude which was purified by silica gel column chromatography (2:8 ethyl acetate-hexane as an eluent) afforded **C** as a white solid. Yield: 3.4 g (27 %); ^{1}H NMR (400MHz, $CDCl_3$): δ (ppm) 7.67 (t, *J* = 9.6Hz, 2H), 7.22 (d, *J* = 2.4Hz, 1H), 7.08 (dd, *J* = 2.4Hz, *J* = 8.8Hz, 1H), 6.99-6.97 (m, 2H), 6.02 (s, 1H), 5.31 (s, 2H), 3.56 (s, 3H). ^{13}C NMR (100 MHz, $CDCl_3$): δ (ppm) 155.6, 154.2, 135.9, 129.8, 129.6, 125.1, 116.7, 116.2, 109.2, 108.9, 94.6, 56.5.

Compound D. To a solution of **C** (3.39 g, 16.6 mmol) in DMF (30 mL) was added K_2CO_3 (3.44 g, 24.91 mmol) and allowed to stirring for 30 min under nitrogen atmosphere. To this solution iodomethane (1.61 mL, 24.91 mmol) was added slowly. The reaction mixture was stirred for additional 1h at room temperature. The solvent was removed *in vacuo*. The residue was dissolved in ethyl acetate

and washed with brine solution (2 x 50 mL). The organic layer was dried over Na_2SO_4, and concentrated under reduced pressure to obtained the crude which was further purified by column chromatography (2:8 ethyl acetate-hexane as an eluent) afforded **D** as a colorless dense liquid. Yield: 3.5g (97 %); ^{1}H NMR (400MHz, $CDCl_3$): δ (ppm) 7.68 (t, *J* = 8.6Hz, 2H), 7.34 (d, *J* = 2.4Hz 1H), 7.10 (d, *J* = 2.4Hz 1H), 7.08 (t, *J* = 2.4Hz, 1H), 7.04 (dd, *J* = 2.4Hz, *J* = 8.8Hz, 1H), 5.30 (s, 2H), 3.91 (s, 3H), 3.54 (s, 3H). ^{13}C NMR (100 MHz, $CDCl_3$): δ (ppm) 158.3, 155.8, 135.9, 129.3, 129.2, 125.1, 116.9, 116.5, 109.5, 105.5, 94.7, 56.4, 55.6.

Compound E. To a solution of **D** (3.5 g, 16.06 mmol) in anhydrous diethyl ether (40 mL) was added *tert*-butyl lithium (23.37 mL, 24.1 mmol) dropwise over a period of 30 min at -20 ^{0}C under nitrogen atmosphere. The resulting mixture was allowed to stirring at same temperature for 60 min and anhydrous DMF (1.85 mL, 24.08 mmol) was added to this reaction mixture over a period of 30 min and the whole reaction mass was allowed to stirring for 1 h under nitrogen atmosphere. The reaction mass was poured into ice water and extracted with ethyl acetate. The organic layer dried over Na_2SO_4 and distilled out the organic solvent to obtain the crude which was purified by column chromatography (2:8 ethyl acetate-hexanes as an eluent) afforded **E** as a colorless dense liquid and upon standing it was transformed into white solid. Yield: 2.25g (57 %); ^{1}H NMR (400MHz, $CDCl_3$): δ (ppm) 10.53 (s, 1H), 8.30 (s, 1H), 7.75 (d, *J* = 8.8Hz, 1H), 7.37 (s, 1H), 7.05-7.03 (m, 2H), 5.39 (s, 2H), 3.91 (s, 3H), 3.56 (s, 3H). ^{13}C NMR (100 MHz, $CDCl_3$): δ (ppm) 189.9, 160.5, 156.0, 139.5, 131.7, 129.6, 123.9, 118.4, 109.2, 105.2, 95.0 (t, *J* = 7.6 Hz), 56.8 (d, *J* = 8.3 Hz) , 55.6 (d, *J* = 13.7 Hz).

Compound F. Compound **E** (0.70 g, 2.83 mmol) was dissolved into isopropyl alcohol (15 mL) and 5(M) HCl (7 mL) was added to this reaction solution and allowed to stirring for additional 3h at 65 ^{0}C under nitrogen atmosphere. After cooled to room temperature, isopropyl alcohol was distilled out under reduced pressure. The crude was dissolved in ethyl acetate and washed with brine solution (2 x 30 mL). The organic layer was dried over to Na_2SO_4 and concentrated under reduced pressure to obtained the crude which was purified by column chromatography (2:3 ethyl acetate-hexane as an eluent) afforded **F**

as a yellow solid. Yield: 0.435 g (76 %); ^{1}H NMR (400MHz, $CDCl_3$): δ (ppm) 10.48 (s, 1H), 9.98 (s, 1H), 8.01 (s, 1H), 7.73 (d, J = 8.8Hz, 1H), 7.13 (s, 1H), 6.99 (dd, J = 2.4Hz, J = 8.8Hz, 1H), 6.95 (d, J = 2.8Hz, 1H), 3,93 (s, 3H).^{13}C NMR (100 MHz, $CDCl_3$): δ (ppm) 196.1, 161.4, 156.9, 140.5, 137.6, 131.3, 123.2, 120.5, 118.2, 110.8, 104.4, 55.8.

Compound G. To a solution of Compound **F** (0.305 g, 1.5 mmol) and dimethyl malonate (0.186 mL, 1.62 mmol) in 20 mL ethanol was added piperidine (0.045 mL, 0.45 mmol). The resulting mixture was stirred at 65℃ under nitrogen atmosphere for 2h. The mixture was cooled to room temperature and evaporated under reduced pressure, and the crude product was recrystallized with dichloromethane and hexane to afford compound **G** as brown solid. Yield: 0.353 mg (77 %); ^{1}H NMR (400MHz, $CDCl_3$): δ (ppm) 8.64 (s, 1H), 8.04 (s, 1H), 7.82 (d, J = 8.8Hz, 1H), 7.57 (s, 1H), 7.17-7.12 (m, 2H), 3.97 (s, 6H). ^{13}C NMR (100 MHz, $CDCl_3$): δ (ppm) 151.6, 149.7, 149.1, 138.3, 135.7, 130.9, 130.8, 130.7, 126.1, 120.3, 120.2, 115.9, 111.5, 105.1, 55.9, 53.2.

Compound 2. Compound **G** (0.350 mg, 1.2 mmol) was dissolved into HBr (10 mL) and allowed to stirring for 12h at 120 ^{0}C under nitrogen. After cooling to room temperature, H_2O was added (50 mL) and the precipitate obtained was filtered off, washed with diethyl ether. The residue was digested with boiling MeOH (30 mL) and the insoluble impurities were removed by filtration. The filtrate was evaporated and the crude product was further recrystallized from dichloromethane/hexane to afford compound **2** as a dark yellow solid. Yield: 0.16 g (52.2 %); ^{1}H NMR (400MHz, d_6-DMSO): δ (ppm) 10.41(s, 1H), 8.75 (s, 1H), 8.36 (s, 1H), 7.90 (d, J = 8.8Hz, 1H), 7.61 (s, 1H), 7.17 (s, 1H), 7.11 (d, J = 9.2Hz, 1H). ^{13}C NMR (100 MHz, d_6-DMSO): δ (ppm) 164.6, 159.2, 157.6, 151.4, 149.5, 138.4, 132.1, 131.8, 125.3, 120.1, 117.2, 115.9, 110.2, 108.7.

CMP1. **2** (0.10 g, 0.39 mmol), (2-aminoethyl) triphenylphosphonium bromide (0.166 g, 0.43 mmol) and 1-hydroxybenzotriazole (0.104 g, 0.47 mmol) were dissolved in dry DMF (10 mL) and DIPEA (0.1 mL) was added to the reaction mixture and the whole reaction mass was allowed to stirring at room temperature for 1 h under nitrogen atmosphere. To this mixture, (Benzotriazole-1-yioxy) tripyrrolidino

phosphonium hexafluorophosphate (0.244 g, 0.468 mmol) was added and whole the reaction mixture was stirred for 12 h under dark conditions. The solvent was evaporated and the crude was purified by column chromatography using 10 % methanol in chloroform as an eluent to give **CMP1** as a yellow solid. Yield: 0.158 g (48 %); m.p. 177~179 ^{0}C. ^{1}H NMR (400MHz, d_6-DMSO): δ (ppm) 10.49 (s, 1H), 8.99 (t, *J* = 5.6Hz, 1H), 8.78 (s, 1H), 8.41 (s, 1H), 7.89-7.66 (m, 17H), 7.19-7.13 (m, 2H), 3.90-3.84 (m, 2H), 3.70 (brs, 2H). ^{13}C NMR (100 MHz, d_6-DMSO): δ (ppm) 161.4, 160.1, 158.5, 150.1, 148.4, 148.3, 137.6, 134.7, 133.4 (d, *J* = 9.9Hz), 131.6, 131.1, 130.0 (d, *J* = 12Hz), 124.7, 119.5, 118.5, 117.7, 116.0, 115.2, 109.6, 108.0, 33.3, 21.2. HRMS (FAB$^+$): m/z calcd for $[C_{34}H_{27}NO_4P]^+$: 544.1672, found: 544.1672.

^{1}H-NMR, ^{13}C-NMR and HRMS of **1**, **2** and **CMP1**

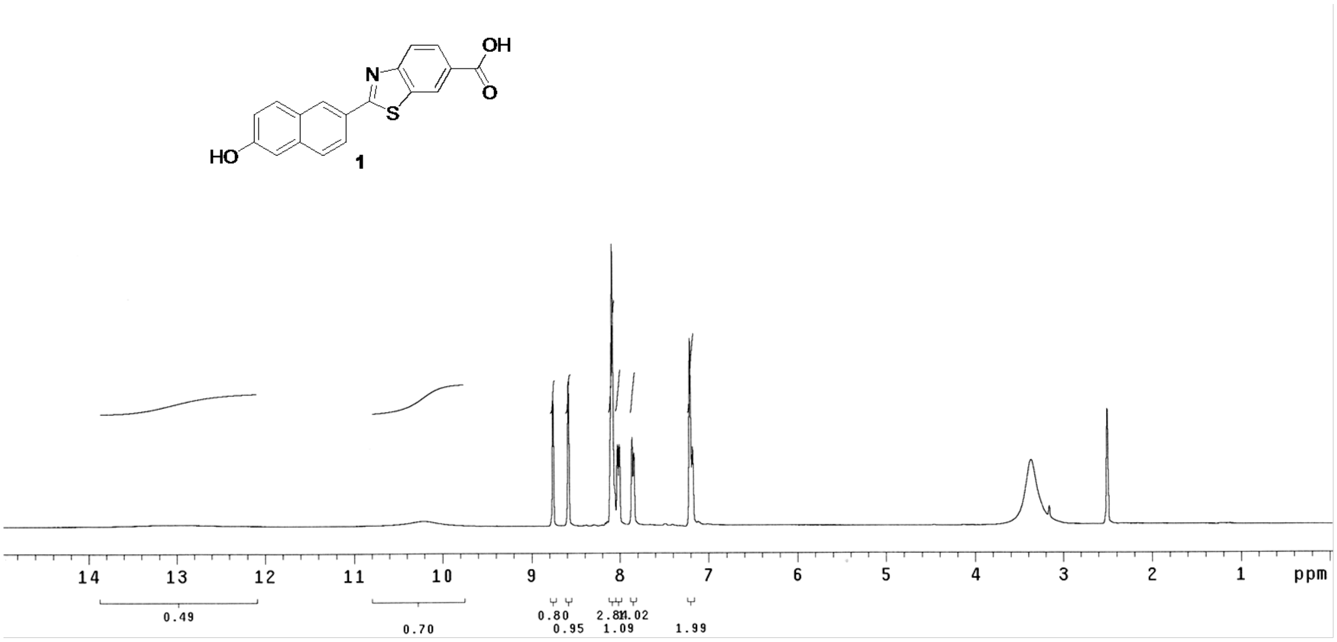

Figure S1. ^{1}H-NMR spectrum (400 MHz) of **1** in d_6-DMSO.

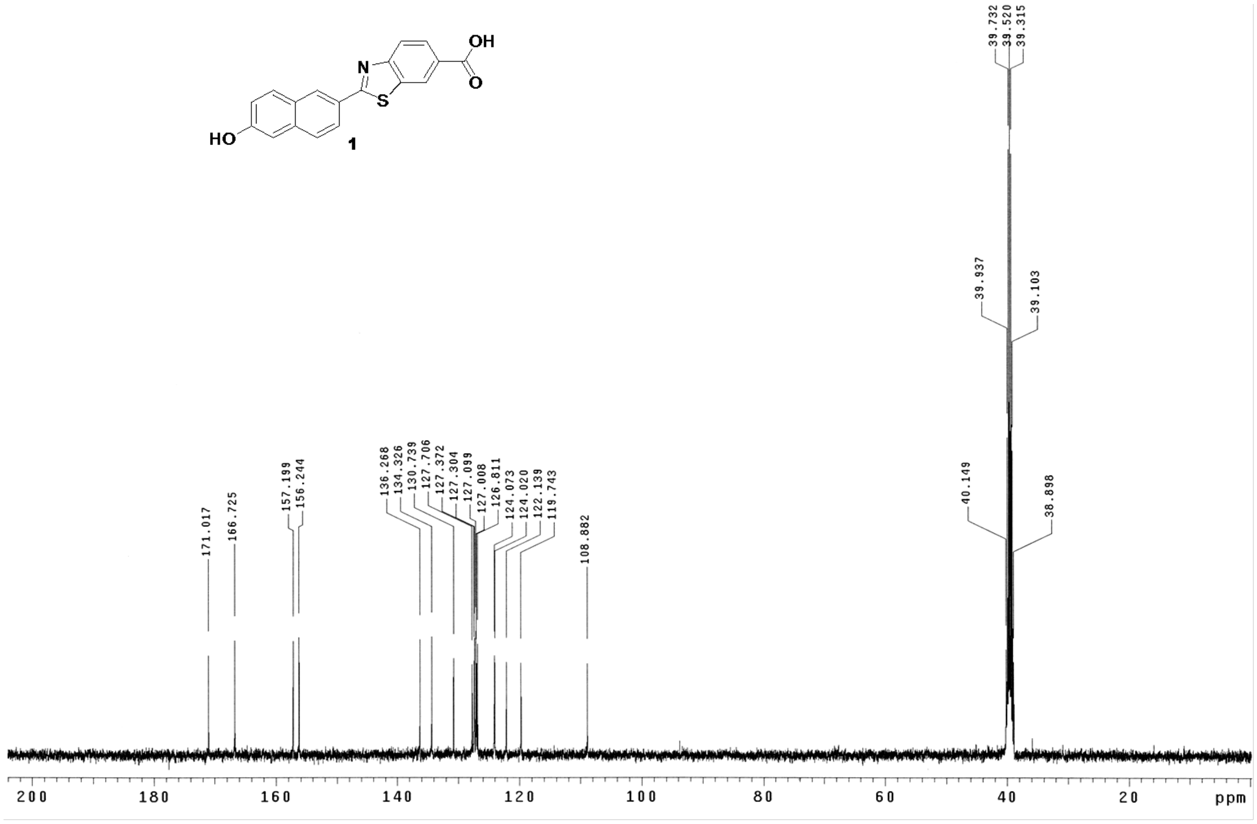

Figure S2. ^{13}C-NMR spectrum (100 MHz) of **1** in d_6-DMSO.

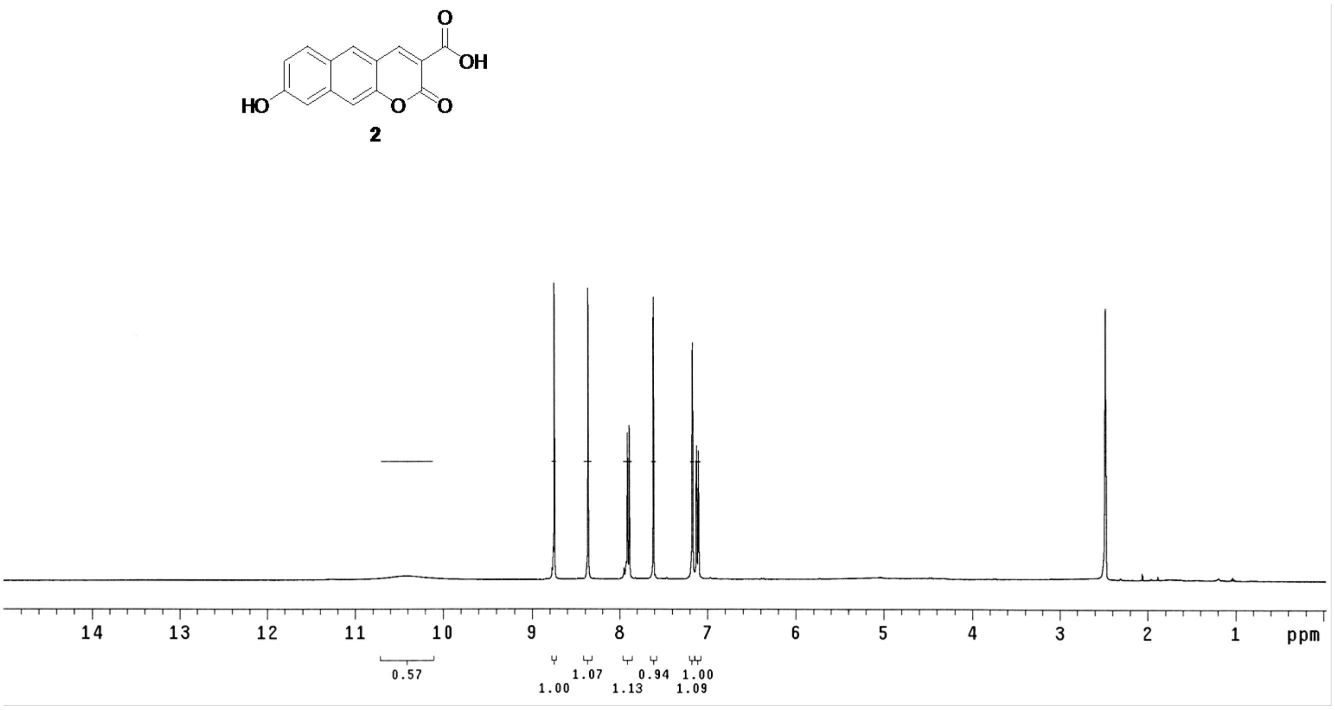

Figure S3. ^{1}H-NMR spectrum (400 MHz) of **2** in d_6-DMSO

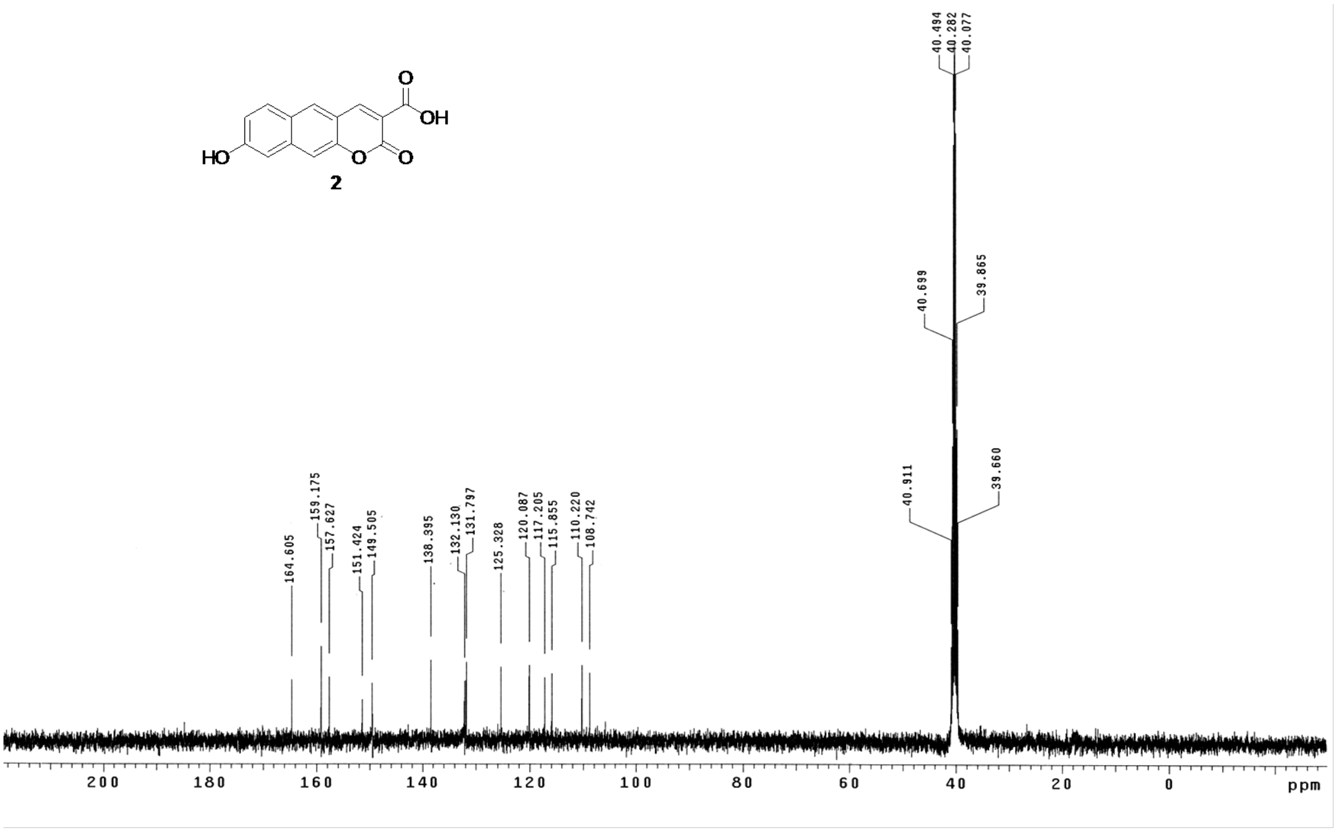

Figure S4. ^{13}C-NMR spectrum (100 MHz) of **2** in d_6-DMSO.

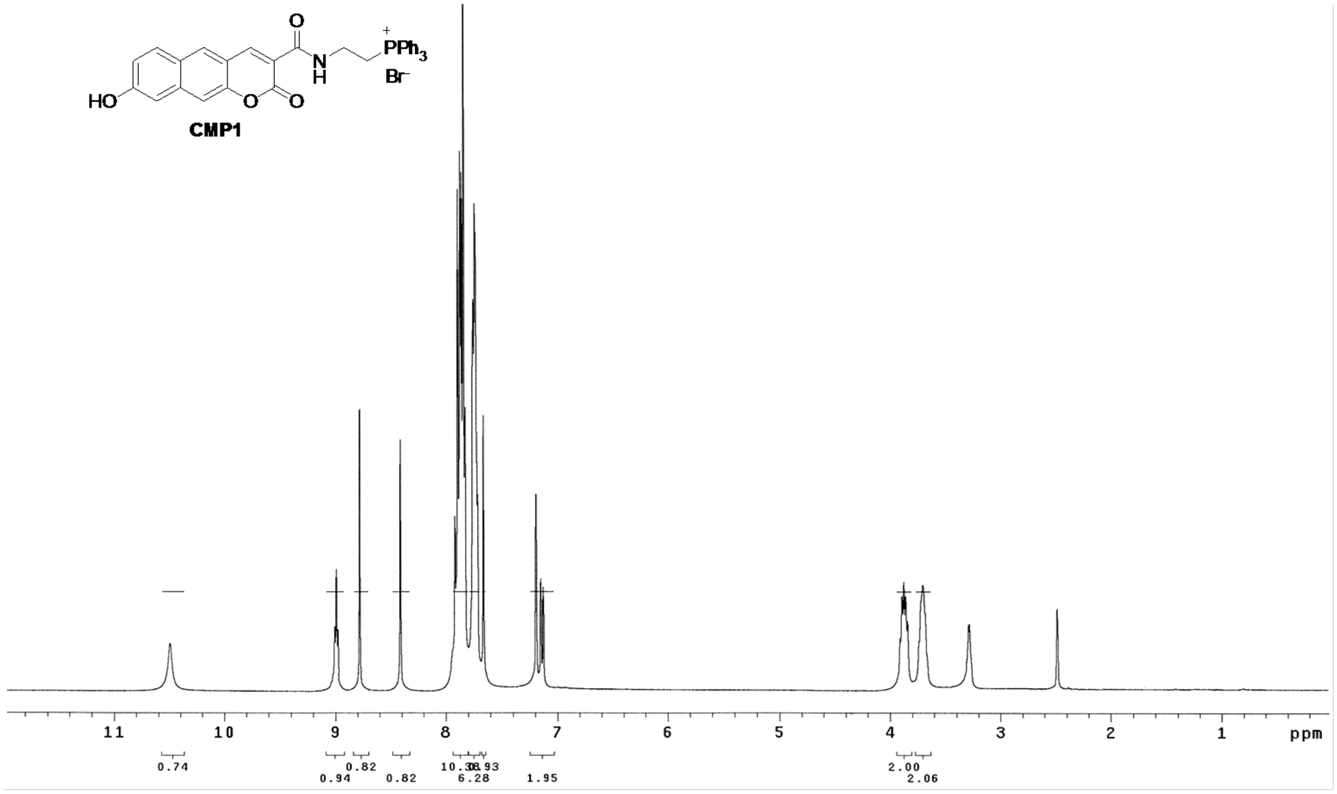

Figure S5. ^{1}H-NMR spectrum (400 MHz) of **CMP1** in d_6-DMSO

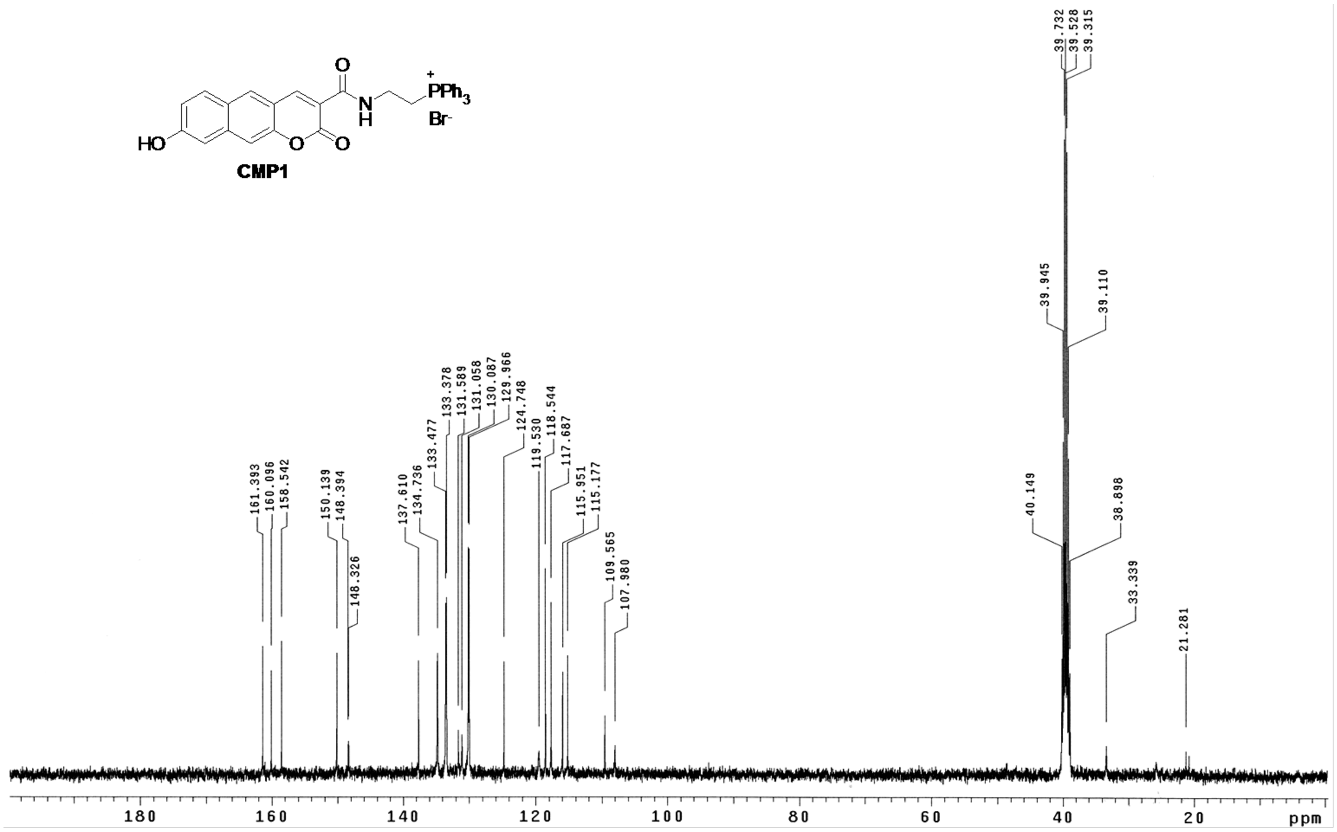

Figure S6. ^{13}C-NMR spectrum (100 MHz) of **CMP1** in d_6-DMSO

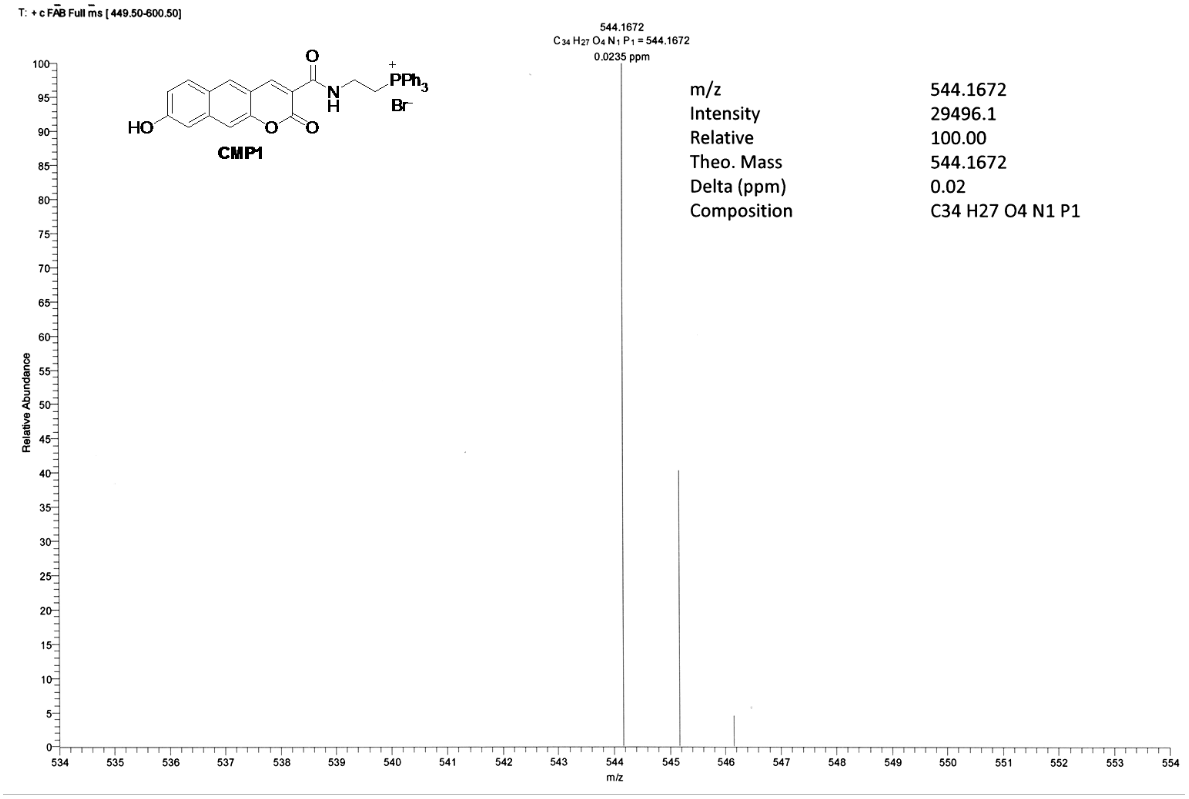

Figure S7. HRMS spectrum of **CMP1.**

pH reversibility of CMP1. To performed the pH reversibility of **CMP1** between pH 5.0 and pH 9.0, the pH solution of **CMP1** (1.0 μM) in buffer (0.1 M KH_2PO_4 buffer) was adjusted back and forth by 1 (N) HCl and 1 (N) NaOH, and then measured by pH-meter (Orion Star A211; Thermo Scientific).

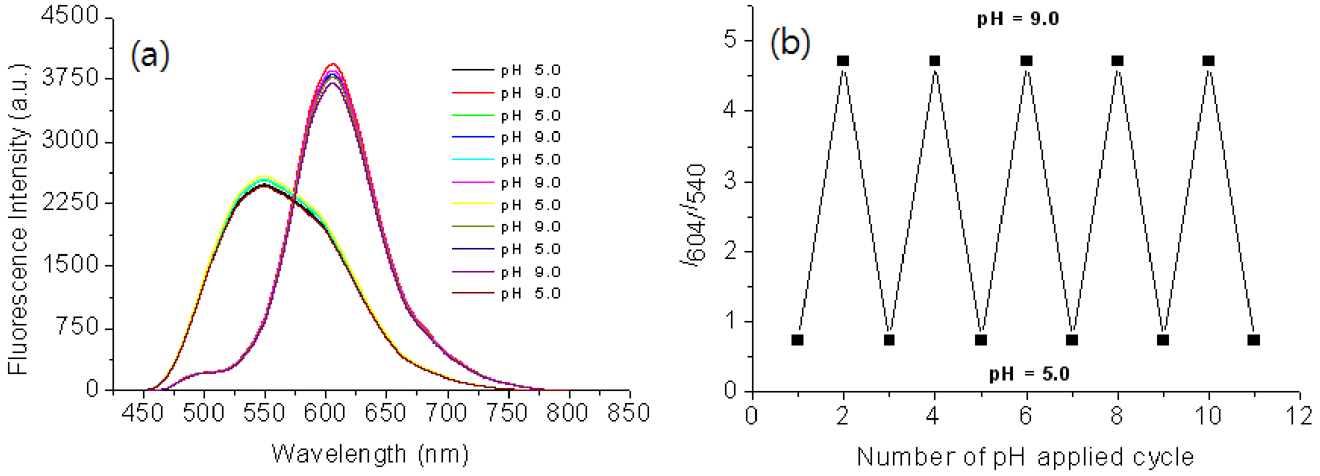

Figure S8. (a) The change in fluorescence emission spectra of **CMP1** with alteration of pH in buffer (0.1 M KH_2PO_4 buffer). (b) Change in fluorescence intensity ratio I_{604}/I_{540} of **CMP1** determined upon accomplished successive pH cycles. The excitation wavelength was 425 nm.

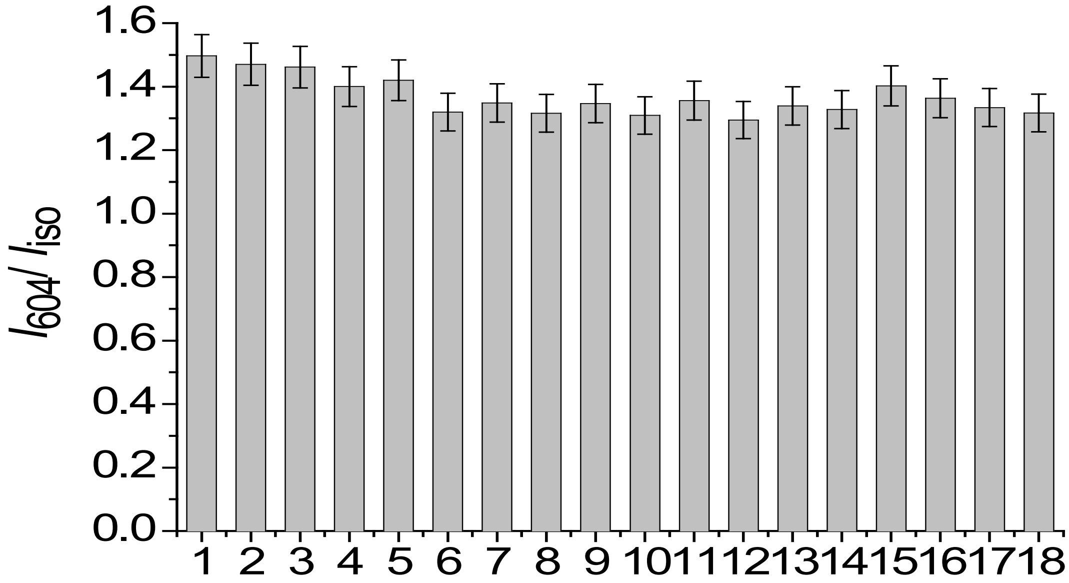

Figure S9. Fluorescence intensity ratio of 1 μM **CMP1** towards different metal ions and biological redox species. 1, probe itself; 100 μM of 2, Na^{+}; 3, K^{+}; 4, Ca^{2+}; 5, Zn^{2+}; 6, Mn^{2+}; 7, Mg^{2+}; 8, Cu^{2+}; 9, Fe^{2+}; 5 mM of 10, GSH; 11, Cys; 12, Hcy; 100 μM of 13, H_2O_2; 14, O_2^{-}; 15, $^{\cdot}OH$; 16, $NO^{\cdot}$; 17, ClO^{-}; 18, Na_2S. Bars represent the ratio (I_{604}/I_{iso} for **CMP1**) of the emission intensities before and after addition of the metal ions and redox species. Data were acquired in KH_2PO_4 buffer (10 mM, pH 7.4). The excitation wavelength was 425 nm.

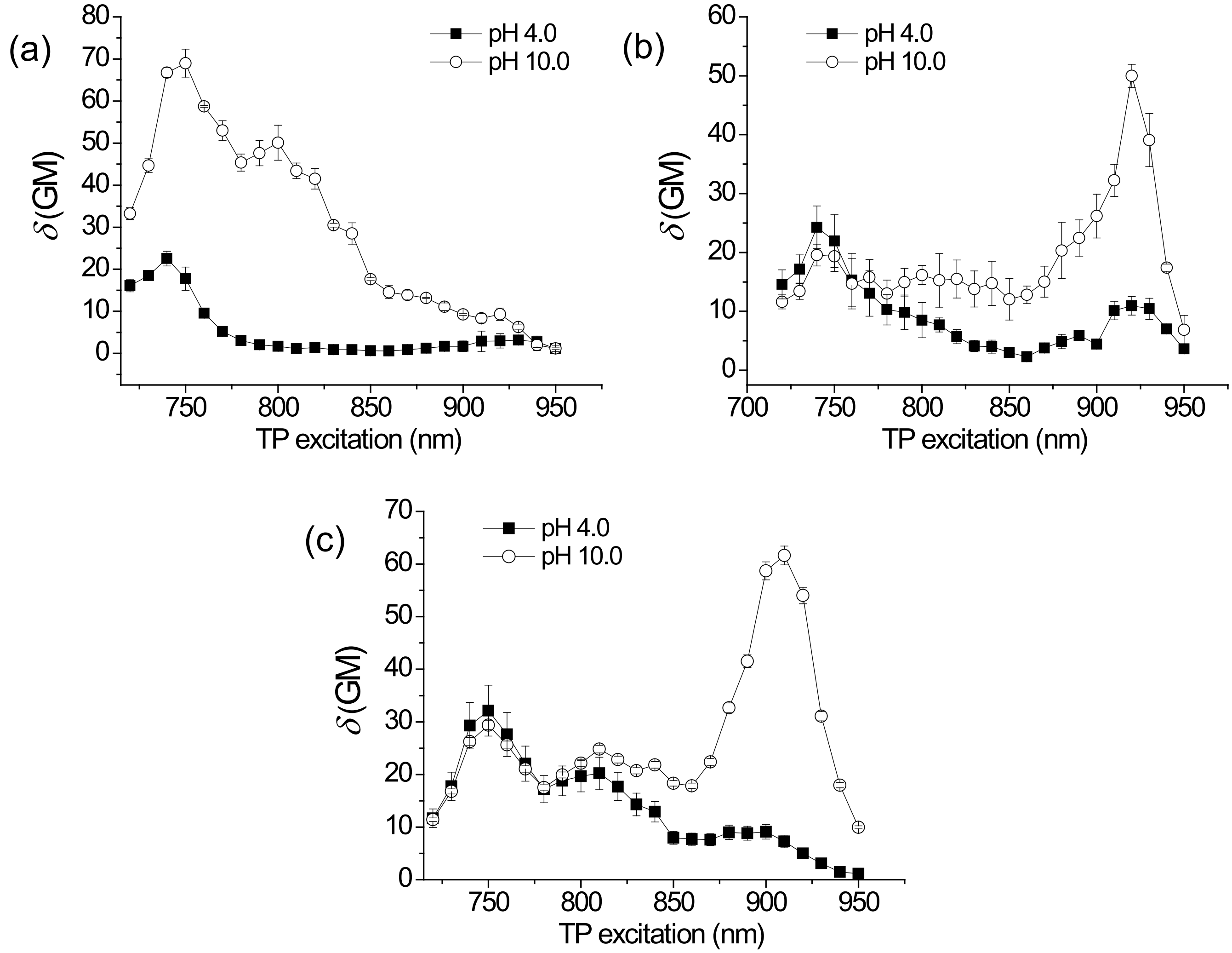

Figure S10. Two-photon absorption cross section spectra of (a) **1**, (b) **2** and (c) **CMP1** in buffer solution at pH 4.0 and pH 10.0, respectively.

Photostability. Photostability of **CMP1** was determined by monitoring the changes in TPEF intensity with time at three designated positions of **CMP1** labeled (2 μM) HeLa cells chosen without bias (Figure S11).

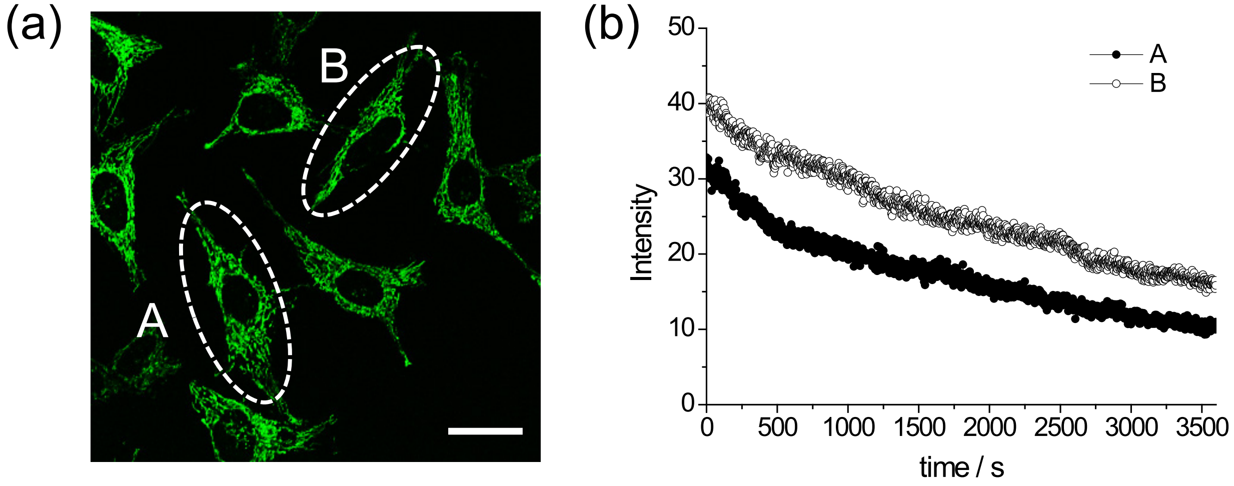

Figure S11. (a) TPM images of **CMP1**-labeled HeLa cells. (b) The relative two-photon excited fluorescence (TPEF) intensity from the A and B of image as function of time. The digitized intensity was recorded with 2.00 sec intervals for the duration of one hour using *xyt* mode. The TPEF intensities were collected 400-650 nm upon excitation at 820 nm for **CMP1** with femto-second pulses. The images shown are representative of the images obtained in the repeat experiments (n = 3). Scale bar: 33 μm.

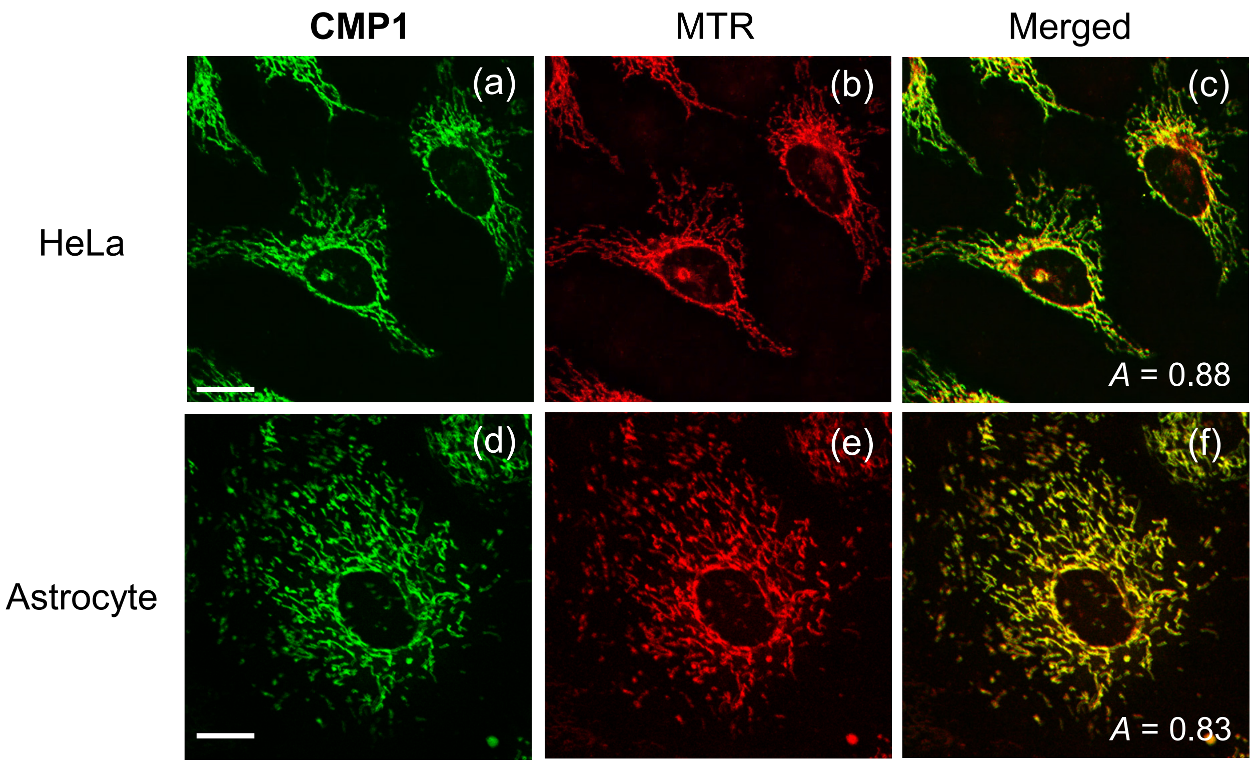

Figure S12. (a,d) TPM and (b,e) OPM images of HeLa cells and astrocyte co-labeled with 2 μM **CMP1** and 1 μM MitoTracker Red FM (MTR). (c,f) Merged image from TPM image of **CMP1** and OPM image of MTR. Excitation wavelength for TPM and OPM are 820 and 552 nm; the emissions were collected at 450-550 nm (**CMP1**) and 650-700 nm (MTR). The images shown are representative of the images obtained in repeat experiments (n = 5). Scale bars: 20 μm.

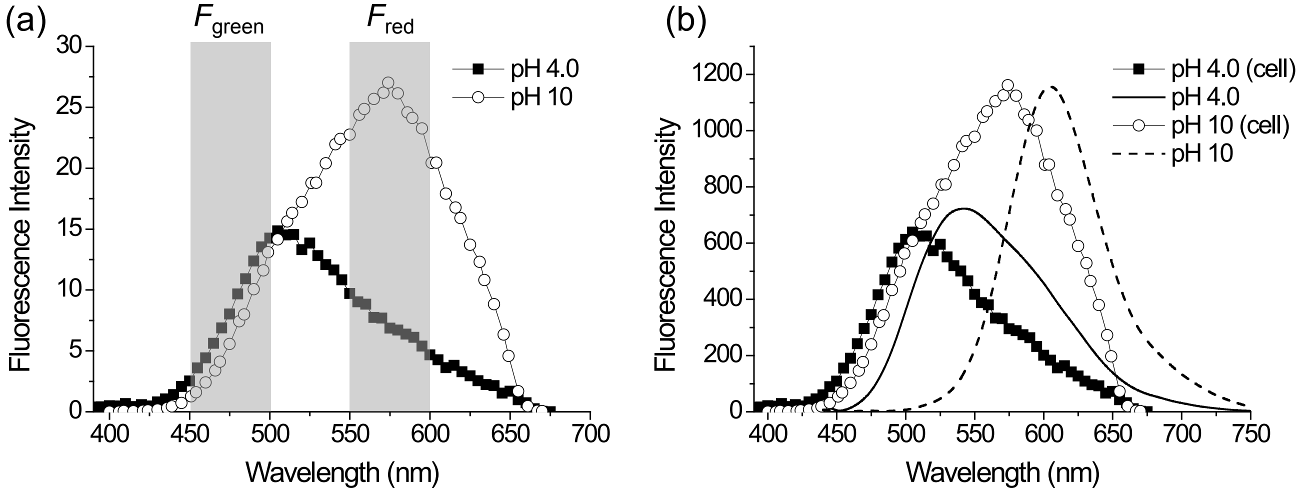

Figure S13. (a) Two-photon excited fluorescence spectra of ionophores-treated HeLa cells labeled with 2 μM **CMP1** at pH 4.0 and pH 10.0. The excitation wavelength was 820 nm. (b) Emission spectra of **CMP1** in buffer solution at pH 4.0 and pH 10.0 and the two-photon excited fluorescence spectra to normalized emission spectra at pH 4.0 (■) and pH 10 (○) acquired from HeLa cells, respectively.

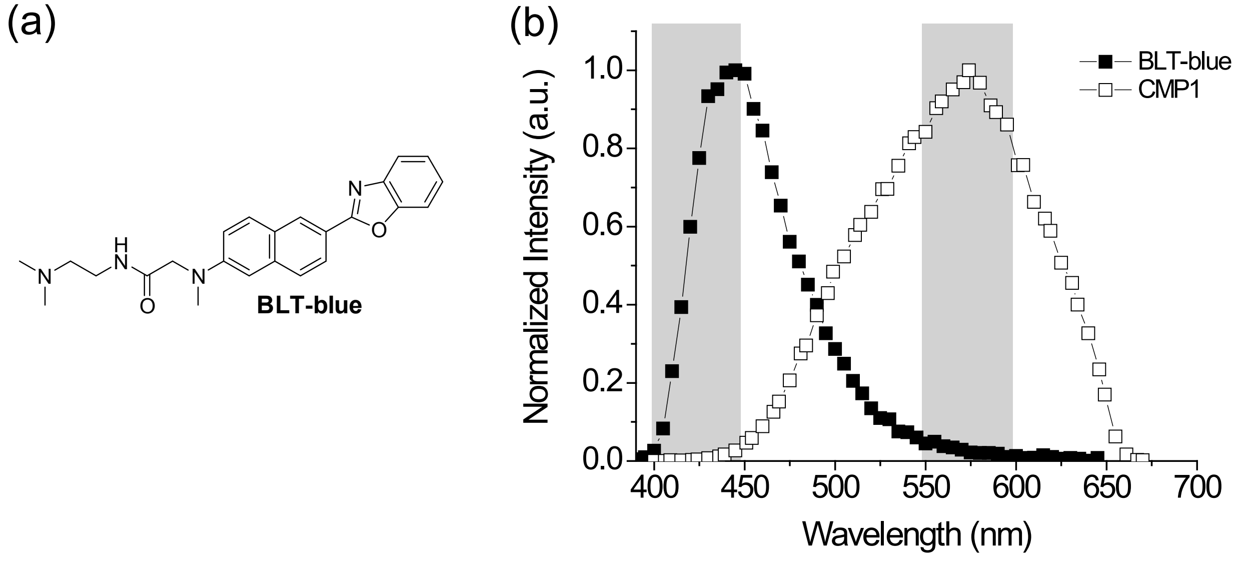

Figure S14. (a) Chemical structure of **BLT-blue**. (b) Normalized two-photon excited fluorescence spectra of **BLT-blue** (■) and **CMP1** (○) acquired from HeLa cells, respectively. The excitation wavelengths were 750 nm and 820 nm for **BLT-blue** and **CMP1**, respectively.

References:

1. C. D. Gabbutt, B. M.Heron, C. Kilner and S. B. Kolla, *Dyes Pigments,* 2012, **94**, 175.

2. K. Serdons, C. Terwinghe, P. Vermaelen, K. V. Laere, H. Kung, L. Mortelmans, G. Bormans and A. Verbruggen, *J. Med. Chem.*, 2009, **52***,* 1428.
